# Automated evaluation of retinal pigment epithelium disease area in eyes with age-related macular degeneration

**DOI:** 10.1038/s41598-022-05006-3

**Published:** 2022-01-18

**Authors:** Naohiro Motozawa, Takuya Miura, Koji Ochiai, Midori Yamamoto, Takaaki Horinouchi, Taku Tsuzuki, Genki N. Kanda, Yosuke Ozawa, Akitaka Tsujikawa, Koichi Takahashi, Masayo Takahashi, Yasuo Kurimoto, Tadao Maeda, Michiko Mandai

**Affiliations:** 1 Kobe City Eye Hospital, 2-1-8 Minatojima Minamimachi, Chuo-ku, Kobe, Hyogo 650-0047 Japan; 2 Department of Ophthalmology and Visual Sciences, Kyoto University Graduate School of Medicine, 54 Shogoin Kawahara-cho, Sakyo-ku, Kyoto, 606-8507 Japan; 3 Laboratory for Biologically Inspired Computing, RIKEN Center for Biosystems Dynamics Research, 6-2-3 Furuedai, Suita, Osaka 565-0874 Japan; 4 Epistra Inc., 2-2-15 Hamamatsu-cho, Minato-ku, Tokyo 105-0013 Japan; 5 VCCT Inc., Kobe Eye Center 5F, 2-1-8 Minatojima-minamimachi, Chuo-ku, Kobe, Hyogo 650-0047 Japan

**Keywords:** Eye diseases, Medical research, Imaging

## Abstract

The retinal pigment epithelium (RPE) is essential for the survival and function of retinal photoreceptor cells. RPE dysfunction causes various retinal diseases including age-related macular degeneration (AMD). Clinical studies on ES/iPS cell-derived RPE transplantation for RPE dysfunction-triggered diseases are currently underway. Quantification of the diseased RPE area is important to evaluate disease progression or the therapeutic effect of RPE transplantation. However, there are no standard protocols. To address this issue, we developed a 2-step software that enables objective and efficient quantification of RPE-disease area changes by analyzing the early-phase hyperfluorescent area in fluorescein angiography (FA) images. We extracted the Abnormal region. This extraction was based on deep learning-based discrimination. We scored the binarized extracted area using an automated program. Our program’s performance for the same eye from the serial image captures was within 3.1 ± 7.8% error. In progressive AMD, the trend was consistent with human assessment, even when FA images from two different visits were compared. This method was applicable to quantifying RPE-disease area changes over time, evaluating iPSC-RPE transplantation images, and a disease other than AMD. Our program may contribute to the assessment of the clinical course of RPE-disease areas in routine clinics and reduce the workload of researchers.

## Introduction

Retinal pigment epithelial (RPE) cells are essential for the function and maintenance of photoreceptor cells^1^. Dysfunction of RPE cells often leads to visual impairment which includes age-related macular degeneration (AMD), choroideremia^2^, and retinitis pigmentosa with a mutation in retinoid cycle-related genes such as *RPE65*^3^, *LRAT*, *BEST1*, or phagocytosis genes such as *MERTK*^4^. AMD is the leading cause of vision loss among the older population in advanced countries. AMD is subdivided into two types: neovascular and non-neovascular, typically with geographic atrophy (GA). For neovascular AMD, an intravitreal injection of anti-VEGF agents, photodynamic therapy, or a combination of both are currently the major treatment options for suppressing choroidal neovascularization (CNV)^5,6^. However, anti-VEGF therapy often requires continual injections over the years due to frequent recurrences of CNV. A patient’s vision cannot be easily maintained in the real world^7^. Furthermore, even when the disease-causing CNV is in remission with treatment, persistent RPE atrophy often affects post-treatment vision. Therefore, compensation for impaired RPE by transplantation is an ideal therapeutic approach for both types of AMD, in addition to surgical removal of CNV in some eyes with neovascular AMD^8–11^.

Fundus autofluorescence (FAF) examination and fluorescein angiography (FA) are routinely conducted clinical examinations to evaluate the area of diseased RPE. FAF can detect the distribution of lipofuscin (a metabolite from the digestion of outer photoreceptor segments by RPE cells). The absence of autofluorescence indicates the loss of either RPE or photoreceptors^12^. In FA, intravenously injected sodium fluorescein dye binds to serum albumin and visualizes blood vessels and vascular lesions as hyperfluorescent regions. Findings are termed pooling, leakage, or staining according to the pathological features of the disease site^13^. The "window defect" is another characteristic finding on FA. It is named for a choroidal background fluorescence that becomes evident in the absence of healthy RPE. This appears from the very early-phase of FA examination. The other findings of pooling, leakage, or staining generally become more evident in later phase FA.

To date, we have conducted clinical studies using induced pluripotent stem cell-derived RPE (iPSC-RPE) cells for transplantation in eyes with neovascular AMD. To directly assess the results of RPE transplantation, it is mandatory to quantitatively evaluate the increase or decrease in the RPE-disease area in vivo. FA provides more detailed information on the pathological features of the lesion site compared to FAF. iPSC-RPE transplanted cells often appear dark compared to the RPEs in the healthy fundus on FAF images because of the loss of overlying photoreceptors. Therefore, we previously used FA images to assess iPSC-RPE graft cell survival instead of FAF^8^. We conducted a quantitative evaluation of early-phase abnormal hyperfluorescent areas. We concentrated on the abnormal region by focusing on RPE atrophy or window defects that often precede or include other pathological events or components such as CNV and fibrosis. These areas are illustrated by pooling, leakage, or staining. We showed that the RPE-disease area (abnormal hyperfluorescent area) was reduced after iPSC-RPE transplantation by manually processing the early-phase FA images to binary images for quantitative evaluation^8^. In order to refine this evaluation process, it should include both the annotation of the overall abnormal region and the evaluation of the degree of mottled hyperfluorescence within that region. Because of this rather complicated procedure, similar manual work would be laborious when the sample number increased. It could risk subjective bias contamination.

Recent advances in image analysis technology, including machine learning-enabled development of various software programs, can identify and quantify the lesion area or automate the comparison of a lesion site at various time points. These advances include software that recognizes and characterizes corneal lesions to assist in diagnosis^14^, automatic detection of non-perfusion areas in diabetic macular edema^15^, segmentation of AMD on OCT images^16,17^, and the classification of AMD disease grades^18,19^. However, there have been no reports of automatic or quantitative comparisons of hyperfluorescent areas using FA images. The reasons for these difficulties were that the pathological hyperfluorescent area on the FA image was speckled, the boundaries were unclear, the various FA images had a luminance slope, and the other FA images were rotated, tilted, and enlarged. These difficulties had to be overcome to develop effective software for lesion identification and quantitative comparison using routine clinical images.

This study aimed to propose and demonstrate an efficient, reproducible, and objective analysis method to quantitatively assess the temporal changes in the RPE-disease areas, as determined by abnormal hyperfluorescent areas in FA images. They evaluate the disease progression and efficacy of RPE cell replacement therapy.

To achieve this goal, annotation of hyperfluorescent regions for machine learning is considered to be a basic method. However, annotation of hyperfluorescent regions is difficult due to unclear boundaries and various degrees of atrophy within the lesion. Therefore, we decided to take a two-step approach. This included the prediction of the overall abnormal region by machine learning methods followed by automated binarization within that region.

We used U-net^20^, one of the machine learning methods used for image segmentation tasks, to predict the "Abnormal region" in each extracted FA image. To filter out unnecessary regions (e.g., regions with no abnormalities or blood vessels), we used these predicted regions as masks. We compared the paired FA images of the same eye. The corresponding points were selected and adjusted in size and position, and the summative "Mask area" of both images was uniformly applied to both images. After luminance correction, the sum of the speckled ”Hyperfluorescent area” (Score) pixel numbers were calculated for each image using a customized binarization process in the extracted common Mask area. To validate our program, we first evaluated the errors of the Scores on the same eyes on the same day to verify the variability in scoring. We then applied the program on the eyes with AMD to compare the Scores on 2 different visits. We also applied it over years to quantitatively evaluate the disease progress. We further applied this method to one of our iPSC-RPE transplantation data cases and to central serous chorioretinopathy (CSC), to show a representative example of how the application of this software would function for other purposes or diseases with RPE-disease areas. In this study, we successfully developed practical software by solving some of the most clinically challenging problems related to image analysis. We accomplished this by combining appropriate existing programs.

## Materials and methods

### Definition of terms

First, this program is designed to quantify changes in the “RPE-disease area” defined as the area of abnormal "Hyperfluorescence" (window defects, leakage, pooling, and staining). Abnormal Hyperfluorescence is mostly equivalent with the "window defect" area in non-vascular pathology. However, it may include leakage, pooling, and staining in neovascular AMD. Since we consider that the region with leakage, pooling, and staining may be included in the "RPE-disease region,” we quantify all of these as Hyperfluorescence. We quantify these areas by using early angiography in order to minimize the effect of the expansion of hyperfluorescence due to leakage.

Terms.

- *Abnormal region* The determination of an Abnormal region includes not only Hyperfluorescent regions (window defects, leakage, pooling, staining, and blood vessels) but also hypo-fluorescent regions (blocking and filling defects).
- *Predicted Abnormal region* The regions generated by the program based on deep learning using an RPE-disease region image annotated by a physician as a training dataset.
- *Mask* The area for filtering unnecessary regions for subsequent analyses. We used the Predicted Abnormal regions as the Mask regions.
- *Hyperfluorescent area* The Hyperfluorescent area on fundus FA images, including normal fluorescent areas (e.g., blood vessels) and the abnormal Hyperfluorescent area (window defect, pooling, leakage, staining) in the Mask. In this study, the Hyperfluorescent area is technically defined by images of < 1 min after fluorescein injection. The early-phase was used to minimize the effect of the expansion of hyperfluorescence due to leakage.
- *Reference alignment image* A fundus FA image that is used as a reference when aligning between two images and provides a Reference score when producing the Growth rate described later.
- *Aligned image* A fundus FA image that is moved to align with the reference image when aligning between two images and provides a Test score when producing the Growth rate described later.
- *Score* Sum of pixel numbers of the Hyperfluorescent area in the binarized image.
- *Reference score* Score calculated from Reference alignment image.
- *Test score* Score calculated from Aligned image.
- *Growth rate* The Growth rate represents the (Test score—Reference score)/Reference score. This indicator shows the percentage increase in the Test score compared to the original Reference score. The numerator indicates the RPE-disease area changes between the two images by subtracting the normal blood vessels.
- *Data processing program* A program that takes two FA retinal images from the same individuals as input, outputs images of the Hyperfluorescent area and calculates the Score and determines the Growth rate.

### Guidelines

This study was approved by the Institutional Review Board of Kobe City Eye Hospital, Kobe City Medical Center General Hospital, and the RIKEN Center for Biosystems Dynamics Research. This study was conducted in compliance with the Declaration of Helsinki. The study protocol and its implementation were approved by the Ethics Committee of Kobe City Eye Hospital (approval number: ezn190203). The committee waived the requirement for informed consent due to the retrospective nature of the study.

### Image acquisition

We used FA images acquired from AMD patients who visited the Kobe City Eye Hospital and Kobe City Medical Center General Hospital between January 2006 and February 2021 using SD-OCT (Heidelberg Spectralis, Heidelberg Engineering, Heidelberg, Germany) at 30°.

Because hyperfluorescent lesions of pooling or leakage often expand with recording time, we focused on the Hyperfluorescent area by selecting the early-phase FA of < 1 min for this study. This showed equalized brightness of retinal vessels and no halos.

### Annotation of Abnormal regions

FA images were annotated independently by two ophthalmologists in charge of the AMD outpatient clinic using ImageJ for a local database. A total of 963 images (202 healthy eyes and 761 AMD eyes) were mutually agreed upon for the accuracy of their clinical labels. We assigned 953 images (202 healthy and 751 AMD eyes) for the training set and 10 disease images for the testing set. Annotated regions were defined as areas that were determined to be Abnormal regions, including abnormal Hyperfluorescent areas and hypofluorescent areas.

### Preprocessing

All the FA images were anonymized. Before using the images in our research, we ensured that the image files did not reveal names, birthdays, or patient IDs.

If the obtained FA image provided information such as the photograph date and time derived from the measuring instrument, the relevant area was trimmed and removed. All images were converted before use to 496 pixels × 496 pixels of 8‐bit tiff images by using the rescale function of the scikit-image without anti-aliasing.

### Extraction of Abnormal regions in FA images using U-net.

We used U-net^20^ to build a deep learning model for Abnormal region extraction. The network was implemented using TensorFlow version 2.4.0. We used a Python program on a computer with an NVIDIA Tesla V100 graphics card. The performance of the developed extraction function was quantified by calculating the sensitivity and specificity of 10 test FA images from patients with AMD. The definitions of sensitivity and specificity were as follows:

Sensitivity = number of true Abnormal pixel counts for the entire image/(number of true Abnormal pixels in the entire image + the number of false normal pixels in the entire image).

Specificity = number of true normal pixel counts for the entire image/(number of true normal pixels in the entire image + number of false Abnormal pixels in the entire image).

### Image alignment to binarization

Image alignment: Corresponding points were automatically detected by using FAST corner detection^21^ and BRIEF features^22^. If corresponding points were not detected, we prepared a CSV file containing each image's corresponding points. The transformation matrix was estimated from the corresponding points by RANSAC estimation^23^. The images were aligned (parallel translation, scaling, rotation, and skew) by an affine transformation.

Brightness correction: All aligned images were Gaussian filtered at σ = 70 pixels. After the background luminance was removed, the image was obtained by dividing the pixel value of the image before filtering by the pixel value after filtering. Histogram matching was performed on the reference alignment images.

Binarization: All brightness-corrected images were binarized with a threshold of 1.2 times the mode value of the pixel value. We chose a value of 1.2 times the threshold because this was a reasonable value when viewing various images.

### Mask generation, overlay on the binarized images, and Score calculation

Mask generation: The prediction Mask of the Abnormal regions was obtained using the discriminator for two FA images to be compared. The pixel values of the Mask images are 1 for the area predicted to be an abnormal area and 0 for the others. Because the obtained Mask image was before position alignment, the Mask image after alignment was obtained by applying the alignment parameter for FA image position alignment. One mask image for the overlay was obtained by performing image processing on all mask images after position alignment.

Mask overlay and scoring: By performing AMD image processing on the FA images after binarization and the Mask image for overlay, we obtained the number of pixels of the Hyperfluorescent area (Score).

### Performance evaluation of the created program

We tested whether the program would output the same Score using two images. These images were from the serial captures, from the same FA examination, with the same Hyperfluorescent area (Score) in the early-phase FA images 45–60 s after injection (n = 53 pairs; 60 images). Images with halos were excluded. We calculated the mean and standard deviation of the ratio of the Scores between the two images.

To test the correlation between the Growth rate and subjective judgment by the ophthalmologists, two ophthalmologists unanimously judged 54 pairs (108 images) in the 21-time course cases by three ranks (approximately the same = Rank 1, slightly worse = Rank 2, much worse = Rank 3). We tested the correlation between the Growth rate outputs by the program and human ratings using a trend test (Jonckheere-Terpstra trend test).

For this data, we used the images acquired between 2006 and 2020 by multiple photographers. The intervals between the two compared images ranged from 44 to 2454 days for Rank 1, 213 to 3209 days for Rank 2, and from 365 to 3248 days for Rank 3.

### Application of the created program

We assessed the temporal changes in the Abnormal area (Mask area) and Hyperfluorescent area by calculating the Growth rate at each follow-up point compared to the earliest visit in the series in four AMD eyes over 10 years.

The Growth rate was also calculated before and after iPS cell transplantation in one eye and two cases of central serous chorioretinopathy (CSC) at two points in two eyes of two patients.

## Results

### Overview of processing

**Figure 1:**
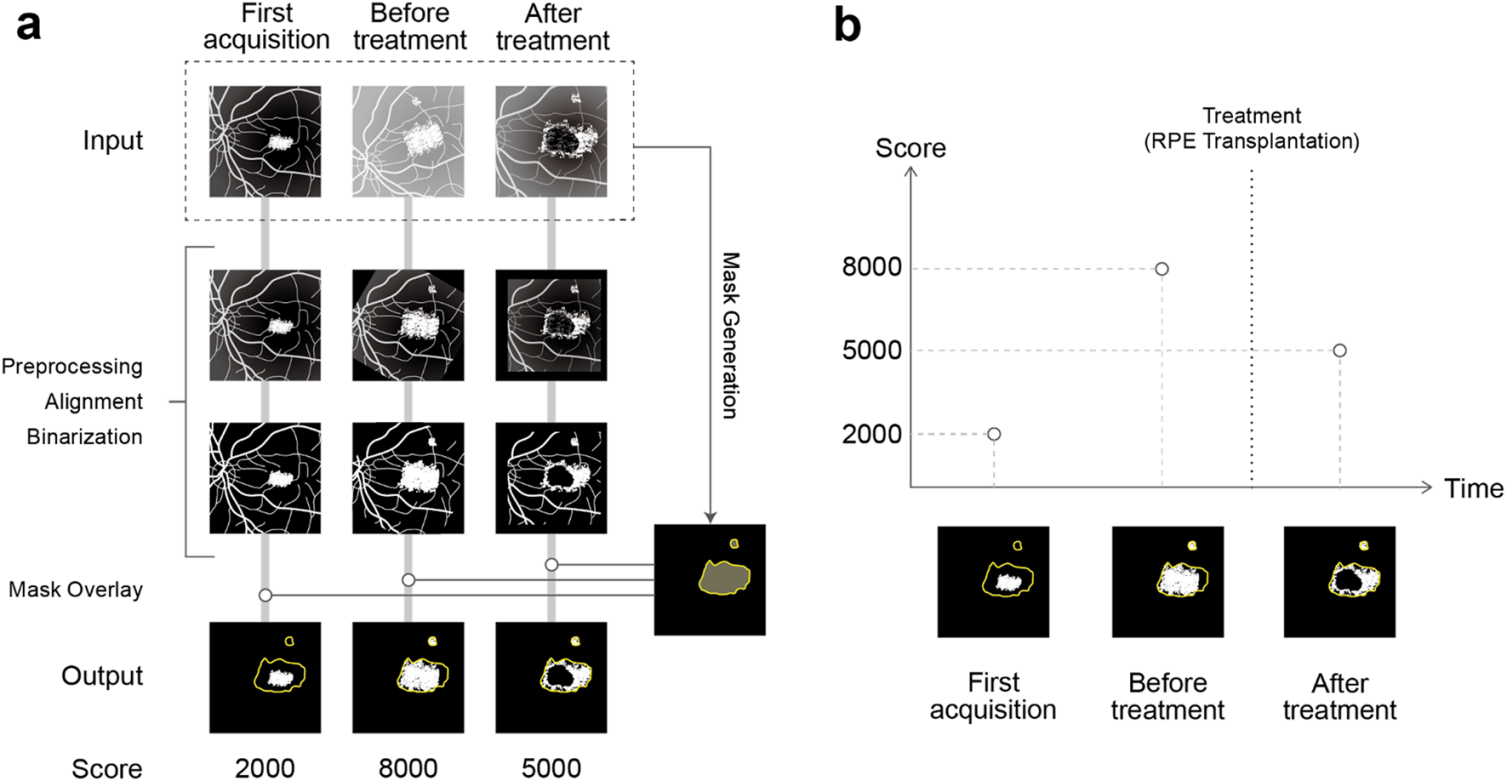
Developed software overview. (**a**) This program outputs Scores by automatically performing the following operations using FA images as inputs: position adjustment, brightness adjustment, binarization, and extraction of Abnormal regions by applying the program obtained by deep learning. (**b**) The developed program automatically calculates a Score based on the Hyperfluorescent area on FA images acquired from a patient over time.

**Figure 2:**
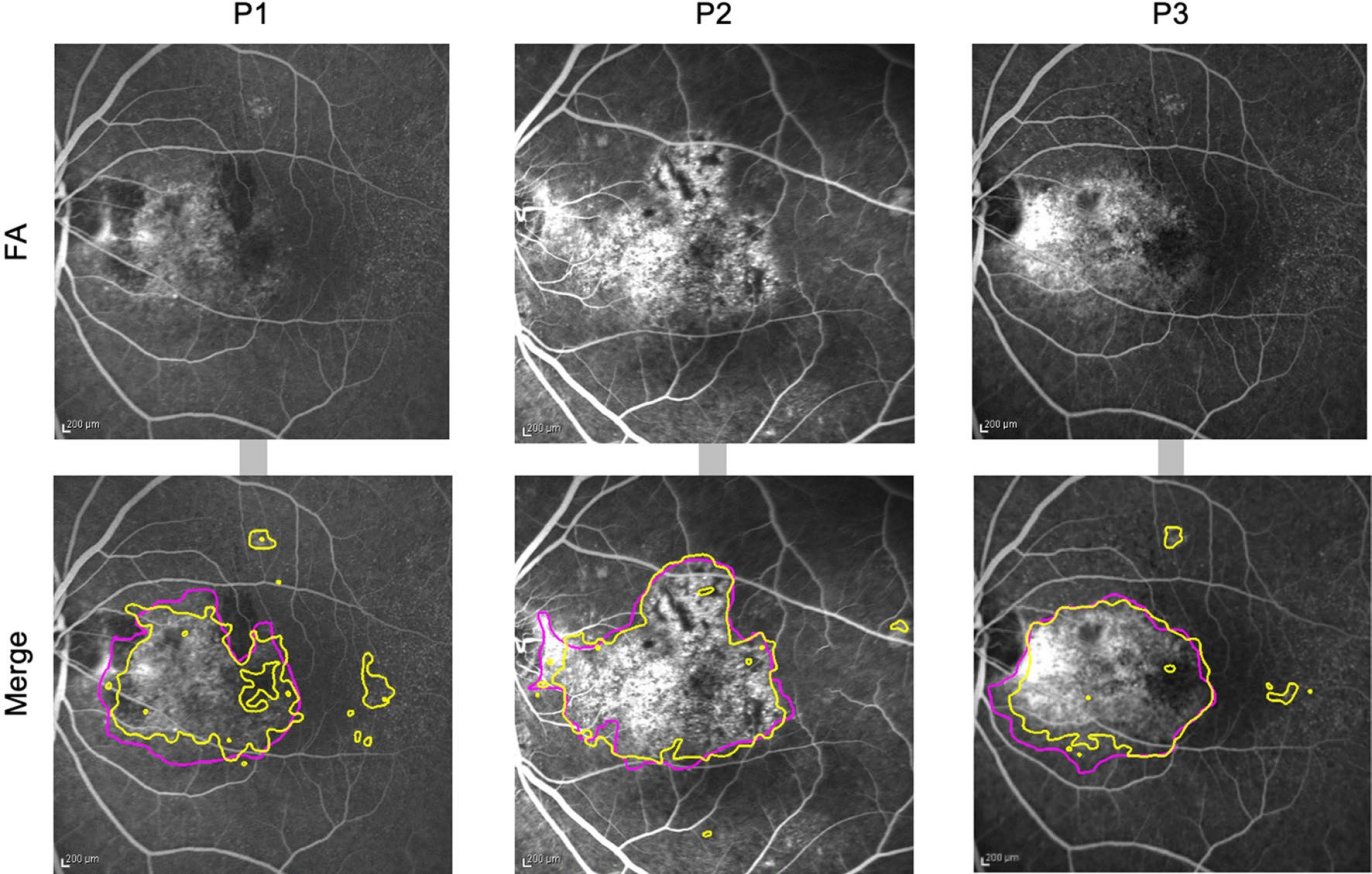
Extraction of abnormal regions from FA images using U-net. Three representative outputs of the processes are based on deep learning (U-net) to detect Abnormal regions (Mask regions) from fluorescein fundus angiography. These images were taken from three different patients (P1-3). Upper: Original raw FA images. Lower: annotated/predicted images. Purple frames represent Abnormal regions annotated by ophthalmologists, and yellow frames represent Abnormal regions predicted by U-net.

**Figure 3:**
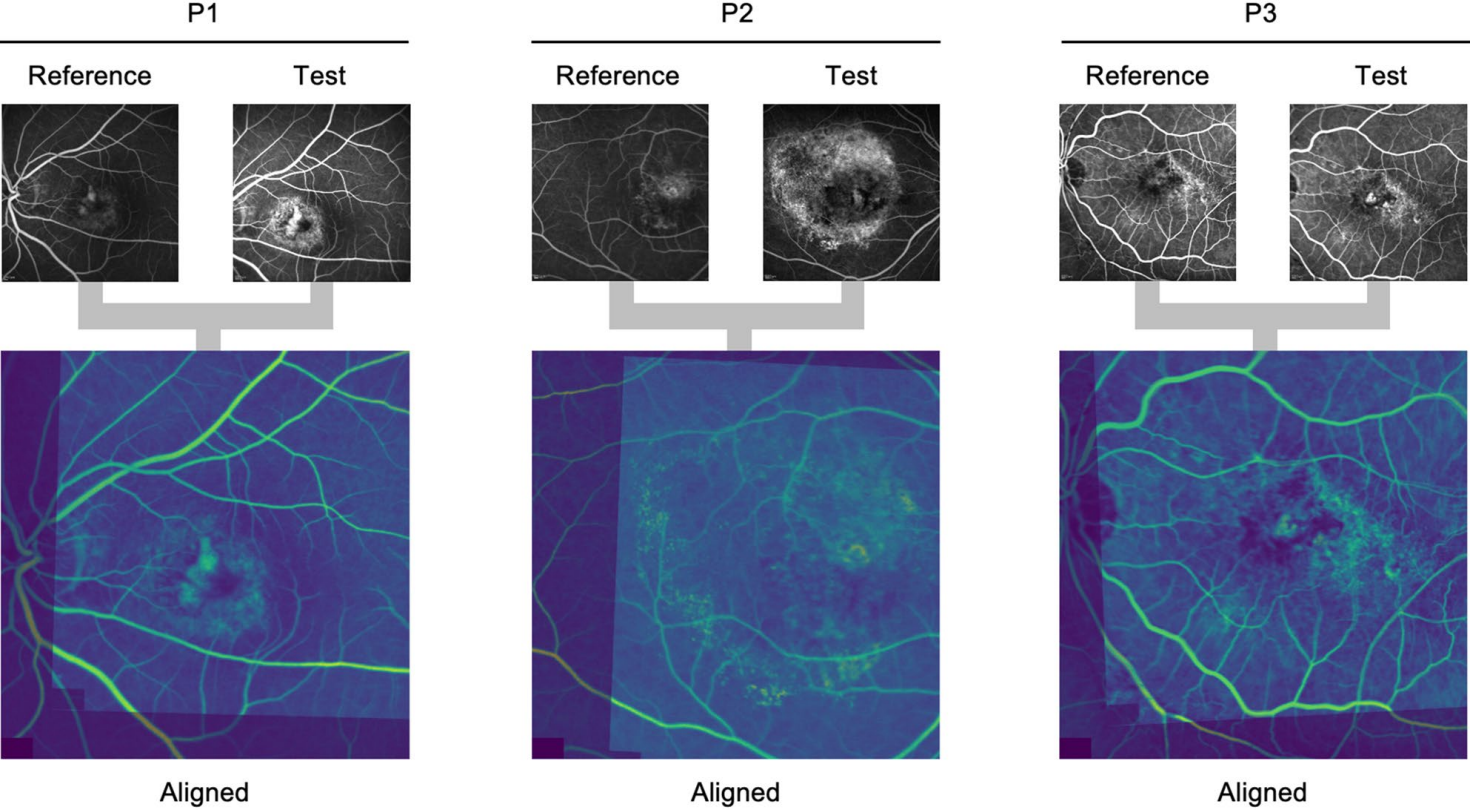
Image alignment. Three representative outputs of the aligned image processes. Upper: A pair of images at different time points of three patients (P1–P3). Lower: Aligned images. We used the FAST corner detection and BRIEF features. The transformation matrix was estimated from the corresponding points by RANSAC estimation. The images were aligned (parallel translation, scaling, rotation, and skew) by an affine transformation.

The data processing program developed in this study outputs Scores by automatically performing the following operations using FA images as inputs: position adjustment, brightness adjustment, binarization, extraction of Abnormal regions by applying the program obtained by deep learning, and Score calculation. An overview of the developed program is shown in Fig. 1. U-net predicted the Abnormal region (Mask region) on each extracted FA image (Fig. 2). We evaluated the performance of the U-net using the loss function. The results were 0.80 for sensitivity and 0.96 for specificity. The Mask regions of the two FA pairs were combined. The corresponding points were selected and adjusted for size and position (Fig. 3). The Score was calculated using a customized binarization process in the combined Mask region. By subtracting the Score of the image pairs, we subtracted the unchanged RPE-disease area, including the normal vessel area. We then calculated the time-varying RPE-disease area.

### Error evaluations of the Scores on the same eyes on the same day

**Figure 4:**
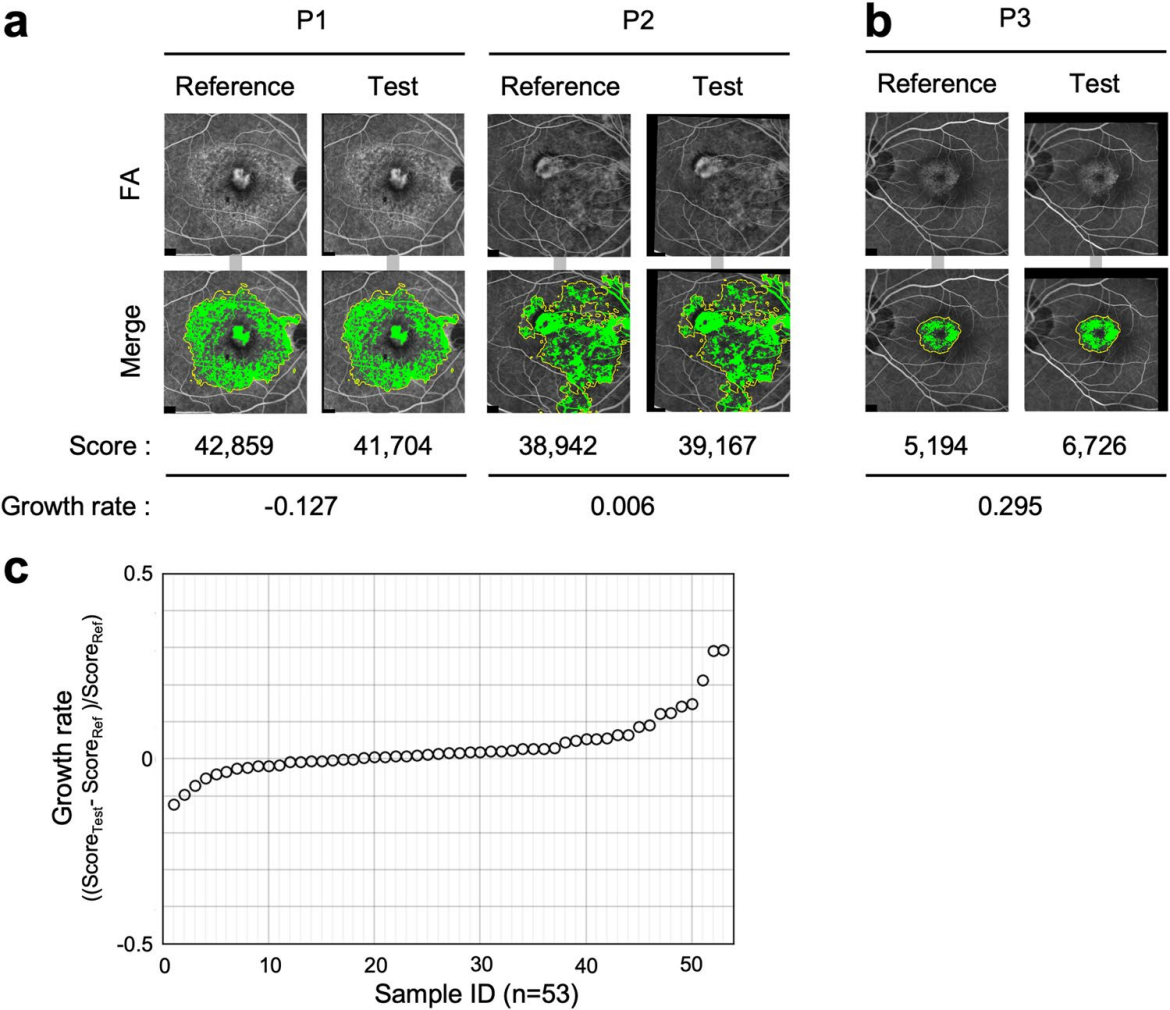
Error validation using the sample FA images from the same eyes. (**a**) A typical pair of images from two patients on the same day. Upper: A pair of FA images that are aligned and brightness corrected by image processing. Lower: Output binary images. Yellow frames represent Abnormal regions predicted by the U-Net. Green areas represent Hyperfluorescent areas. The Score represents the sum of the pixel numbers of the Hyperfluorescent area in the binarized image. The Growth rate is represented as (Test score—Reference score)/Reference score. (**b**) A pair of images with the highest Growth rate. Upper and Lower: represent the same as (**a**). (**c**) Graph showing the Growth rate distribution.

We evaluated the errors of the Scores on two images from serial captures of the same eye (Figs. 4, S1). Figure 4a shows a typical pair of images from the serial captures of two patients on the same day. The image of the later capture in the FA examination was used as the Test score. Figure 4b shows a pair of images with the highest Growth rate. We calculated the distribution of the Growth rates. The mean ± standard deviation was 0.031 ± 0.078 (n = 53). Figure 4c shows the distribution of the Growth rates. The x-axis represents the number of samples and the y-axis represents the Growth rate.

### Comparison of the Scores on the eyes at 2 different visits

**Figure 5:**
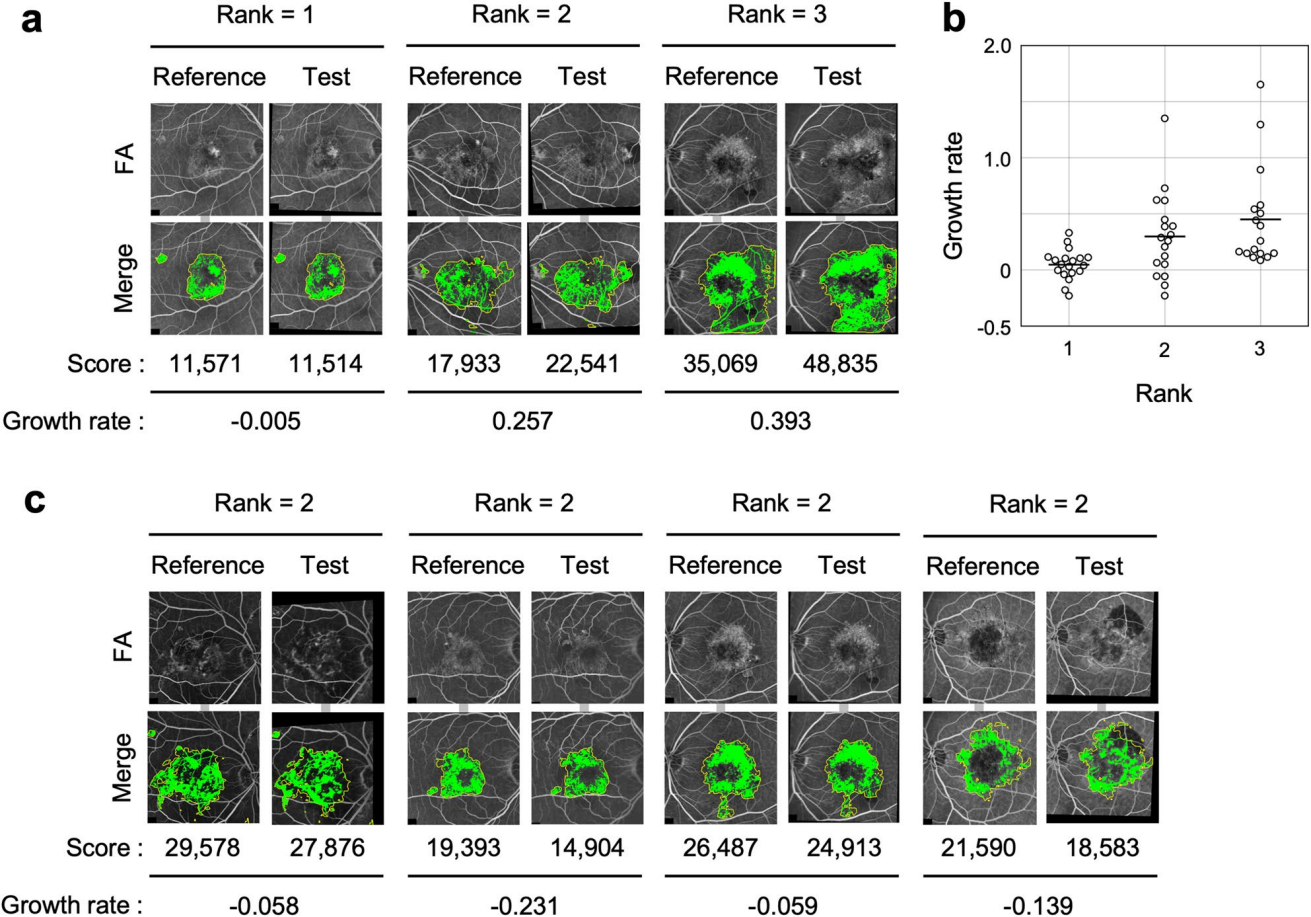
Comparison of Score change with a specialized ophthalmologist. (**a**) Representative pair of images for each rank as unanimously determined by two specialized ophthalmologists. Rank 1: approximately the same; Rank 2: slightly worse; Rank 3: much worse. Upper: Pairs of FA images aligned and brightness-corrected by image processing. Lower: Output binary images. yellow frames represent Abnormal regions (Mask regions) predicted by U-net. Green areas represent Hyperfluorescent areas. The Score represents the sum of the pixel numbers of the Hyperfluorescent area in the binarized image within the Mask area. The Growth rate represents the (Test score—Reference score)/Reference score. (**b**) The scatter and box plot graph shows the Growth rate in the three ranks of changes in the disease status that were judged by two specialized ophthalmologists. The black bars represent averages. (**c**) Four discrepant cases in Rank 2 where the value of the Growth rate was less than 0. Upper and Lower: represent the same as (**a**).

We compared whether the Growth rate was consistent with the subjective evaluation by ophthalmologists. Two ophthalmologists reviewed the images of the disease time course of each eye and unanimously determined the change in the image at each time point as follows: approximately the same = Rank 1, slightly worse = Rank 2, much worse = Rank 3. (Figs. 5 and S2). Figure 5a shows a representative pair of images for each rank. Figure 5b shows the plots of the Growth rate determined by our program for each rank group. The mean and standard deviation of the Scores were 0.048 ± 0.134 for Rank 1 (approximately the same) (n = 19), 0.205 ± 0.375 for Rank 2 (slightly worse) (n = 18), and 0.450 ± 0.447 for Rank 3 (much worse) (n = 17) (Figs. 5a, S2a–c). The value of the Growth rate increased monotonically with increasing rank using the Jonckheere-Terpstra trend test (*P* = 0.00005616). This indicates that the scoring output of our program can estimate the subjective judgment of ophthalmologists.

The presence of a difference between Rank 1 and Rank 2 suggests that data acquisition by different radiographers and at various times may have resulted in acceptable ratings that matched the physician's observations.

Rank 2 contained four pairs with negative values (Fig. 5b). Figure 5c shows all four cases with negative values in Rank 2. These cases included some expansion of hemorrhage, which appears as hypofluorescence, with an overall stable RPE-disease region. This is a limitation of our current program in that it cannot distinguish lesions such as hemorrhage from healthy hypofluorescence. This was confirmed by comparing the Score output with the judgment of ophthalmologists.

### Quantitative assessment of the clinical course of the RPE-disease area

**Figure 6:**
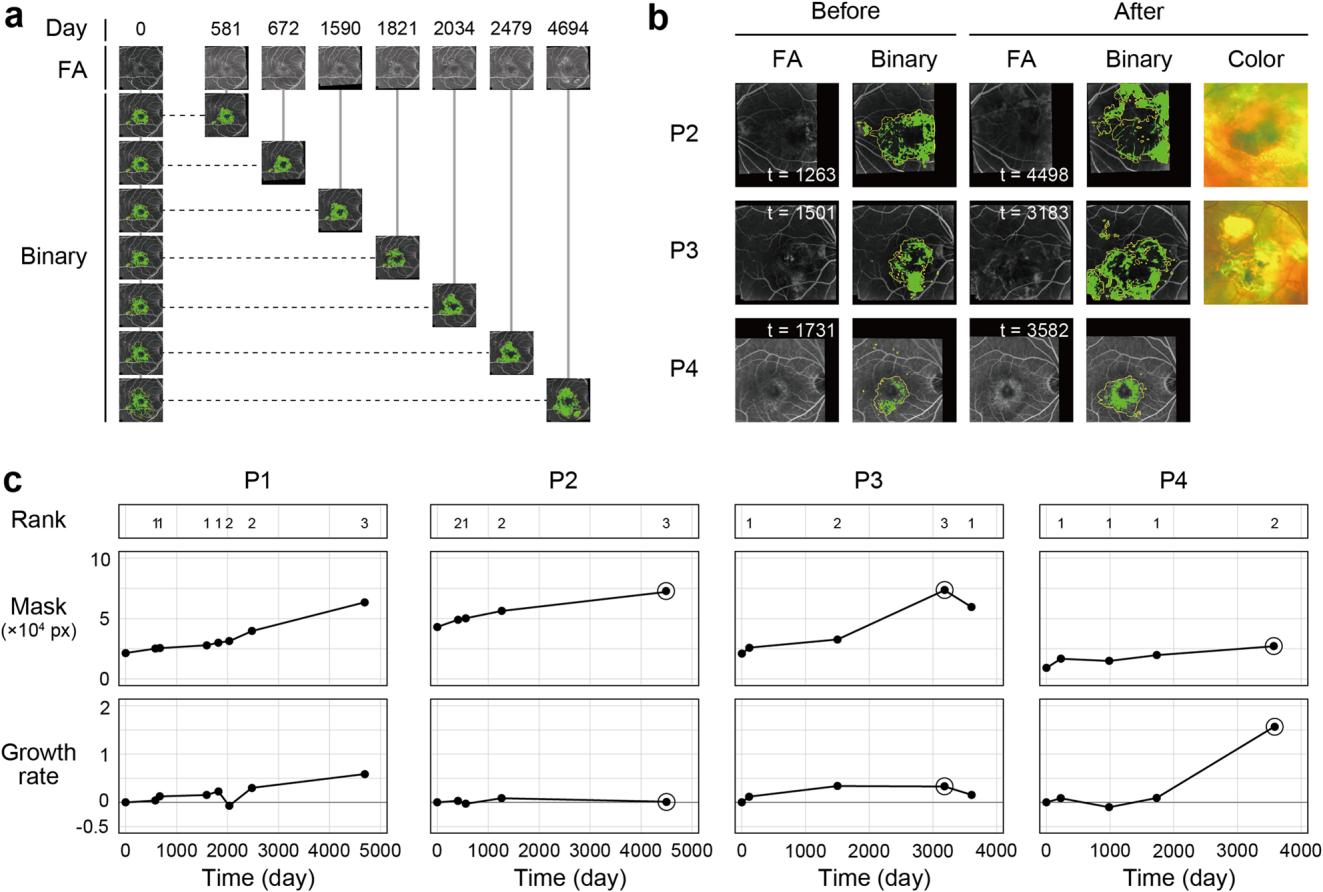
Quantitative assessment of the expanding Hyperfluorescent area over time. Quantitative assessment of the Hyperfluorescent area in four AMD eyes of four patients over 10 years (~ 4694 days). The first image acquisition was defined as day 0. (**a**) Representative temporal analysis of Patient 1 (P1). The FA image of each visit was paired with the initial image, aligned, and corrected for brightness by image processing. Binary images were produced within the merged mask frames of the initial and at each time point as Abnormal regions (yellow frames) predicted by U-Net. Green areas represent areas with Hyperfluorescent Score areas. The Growth rate was calculated between the image of each time point and the initial point. **(b)** Binary images of the indicated time points with a discrepancy between the mask and Growth rate. Rank 3 points with patients 2 and 3 (P2, P3), and Rank 2 points with patient 4 (P4). The relevant sample is shown in the graph in c, with the points circled. **(c)** Temporal change in Growth rate of four eyes and the Mask areas (overall Abnormal areas) at each time point. The judgment by two doctors (Rank 1–3) at each time point is also shown for reference. Images of the circled points are shown in (**b**).

We then applied our program to output the quantitative changes in the RPE-disease area of four eyes that had been followed for over 10 years. These changes were also used as a part of the data in Fig. 5. Growth rate at each indicated time point(t)was calculated in reference to the initial image as day 0 (reference data), as indicated in Fig. 6a with patient 1 (P1). The comparison of the RPE-disease area is primarily for non-exudative eyes, as exudative changes such as hemorrhage and lipids block fluorescence. The Growth rate can underestimate the actual disease growth. Thus, we also plotted the predicted overall Mask area, *that is, the* Mask area at each time point for reference, together with the judgment of the ophthalmologists in Fig. 5 (Rank 1–3) (Fig. 6b). It is noteworthy that the visit with rank 3 in patient 2 and 3 (P2 and 3) showed a discrepancy between the increased Mask area and decreased Growth rate. The FA images of these visits showed blocked fluorescence within the expanding lesion. This was revealed as the transition of the eyes to exudative, active AMD with hemorrhage and lipids (Fig. 6c). In contrast, patient 4 (P4) shows an increase in Growth rate only with a mild increase in the Mask area, as also indicated by the FA images (Fig. 6c, bottom panel). The discrepant point with P1 (t = 2034) is shown in Fig. 5 as one of the four cases with a negative value in Rank 2. These clinical course plots may suggest a different use for this program. It could be used as a disease monitoring program, presenting how the overall disease size changed, as well as indicating an appearance or enlargement of a block lesion including exudative changes, which can be shown by the separation of the Growth rate and Mask area.

### Application of the program to iPSC-RPE cell transplantation and CSC images

**Figure 7:**
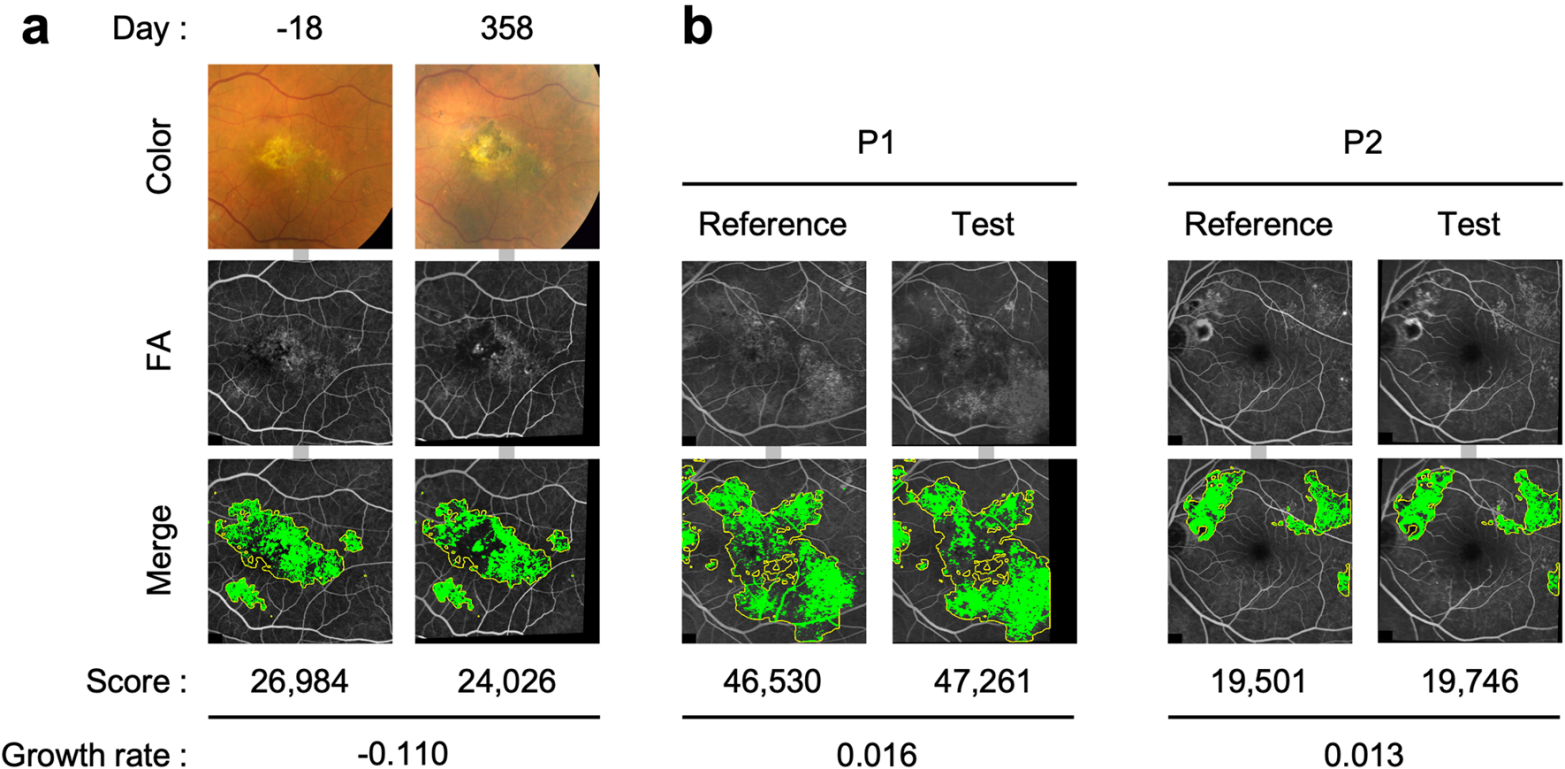
Application of the program to iPSC-RPE cell transplantation and CSC images. (**a**) Quantitative assessment of the Hyperfluorescent areas before and after human iPSC-RPE transplantation in a previously reported case^8^. The date of iPSC-RPE transplantation surgery was defined as day 0. Upper: A pair of fundus color images. The right image shows the black iPS-RPE-transplanted cells at the center of the image. Middle: A pair of FA images aligned, and brightness corrected by image processing. Lower: Binary images. Yellow frames represent Abnormal regions predicted by U-Net. Green areas represent Hyperfluorescent areas. The Score represents the sum of the pixel numbers of the Hyperfluorescent area in the binarized image. The Growth rate represents the (Test score − Reference score)/Reference score. (b) Application of the program to other diseases with Hyperfluorescent areas and central serous chorioretinopathy (CSC). These images were obtained from two different patients (P1-2). Upper: Pairs of FA images aligned and brightness-corrected by image processing. Lower: Output binary images. Yellow frames represent Abnormal regions (Mask area) predicted by U-net. Green areas represent Hyperfluorescent areas. The Score represents the sum of the pixel numbers of the Hyperfluorescent area in the binarized image within the Mask area. The Growth rate represents the (Test score − Reference score)/Reference score.

We applied our program to FA images of the eye obtained before and after iPSC-RPE implantation. We previously reported a similar result using a manual analysis as a part of our clinical study^8^ (Fig. 7a). The Scores before and 1 year after the hiPSC-RPE transplantation were 26,984 and 24,026 respectively (Growth rate, − 0.11). This is an example of the application of our method to evaluate the changes in the mottled area within the overall Abnormal area due to the presence of RPE cells after iPSC-RPE transplantation. We also applied our program to FA images of eyes with CSC. This demonstrates an example of the potential application of our current software for other diseases with RPE atrophy (Fig. 7b). Ophthalmologists judged these images and an increase in the Hyperfluorescent area was observed. The Growth rates were calculated to be 1.016 and 1.013, respectively.

## Discussion

This study developed an efficient, reproducible, and objective analysis method to quantitatively assess temporal changes in the RPE-disease areas as determined by the Hyperfluorescent area in early FA images. The software was used for the comparison of FA images at different visits and quantitative presentation of the long-term clinical course of RPE lesions. Our approach not only provides reproducibility and objectivity, but also shows the possibility of saving ophthalmologists from the burden of reviewing various types of images to judge various levels of change over time in daily clinical practice. It was insufficient to simply apply an existing method to automate the analysis of our clinical FA images. In developing our current method, we successfully solved some of the most clinically challenging problems related to image analysis (discussed below) by combining appropriate existing programs.

A common difficulty with these comparative image analysis is that the images taken at multiple time points have various levels of brightness, contrast, and misalignment. To overcome these difficulties, we used image processing, which includes multiple steps. To extract the feature points, we used the FAST corner detection with vessel branches. We used BRIEF to match feature points and RANSAC to handle translation, scaling, rotation, and skew by aligning without the effect of outliers. We applied a Gaussian filter to address the problem of the luminance slope in the image during image capture. Our program allowed automated processing in seconds and significantly reduced the operational costs of human alignment.

Another difficulty in evaluating the RPE-disease area is that it involves not only the total size of the Abnormal area but also the degree of atrophy inside the lesion. This is indicated by the size and degree of mottled Hyperfluorescence, with undefined boundaries. In the current program, we took a 2-step approach to predict the overall Abnormal area (Mask area) first, followed by the binarization of the mottled area within the Abnormal area. Therefore, we can annotate the disease region in a more precise manner and evaluate the degree of the mottled Hyperfluorescence area. Using Mask, we succeeded in quantifying the difference within the Hyperfluorescent area at two different time points by avoiding the influence of noise or normal hyperfluorescence outside the Mask area (no abnormality region), such as blood vessels. We believe that the comparison within the specified diseased region may beneficially increase the sensitivity to detect subtle changes in pathological hyperfluorescence. We expect the remaining blood vessels inside the Mask area to be voided by subtraction between the two images. We used out-of-mask avoidance and inside-mask cancellation to reduce the risk of overscoring from blood vessels. (In the future, it might also be possible to remove blood vessels without subtraction by using previous reports on extracting blood vessel regions in fundus images^24^).

In this study, the Growth rate of the RPE-disease area calculated by our 2-step program that included two features of size and mottled degree of Hyperfluorescence was well correlated with human judgment. However, the major concern is that the Growth rate based on the Hyperfluorescence area has a risk of underestimating the progressive lesion when the lesions include hypo-fluorescent exudative changes such as hemorrhage and lipids. As shown in Fig. 6, the temporal plots of Mask size and Growth rate presented a discrepancy in these parameters at the point of disease worsening with exudative changes. These combined assessments, or an additional quick observation of color fundus images, can easily overcome the misjudgment by Growth rate. With this possible risk in mind, temporal disease monitoring by Growth rate may also help clarify the disease progress in a quantitative manner.

With all these technological advances in our program, a limitation in the binarization method remains. This can be easily affected by light exposure and results in misjudgment of RPE-disease areas as determined by the Hyperfluorescent area. In the present study, we avoided this risk by excluding images such as those containing halos. It is important to set a standard for the use of adequate images for analysis.

In this study, in addition to AMD, we also applied the method to CSC, which is also associated with RPE-disease. This method may be applicable to eyes with other diseases associated with RPE disorders. As for an additional possibility for the use of the current program, if we compare the early and late-phase FA images from a single examination, we could quantify the degree of leakage, which may reflect the activity of choroidal neovascularization. Although the output Scores based on the FA Hyperfluorescence area seemed to be a good biomarker for the progression of the disease stage and examining the indications and effects of treatment, the quantitative verification of the association between these Scores, FA hyperfluorescence, and visual function should be further investigated. Finally, this was our first challenge using FA images. We may also be able to apply similar technology to the comparison of other grayscale images, including fundus autofluorescence.

## Conclusion

This study developed software for the quantitative assessment of temporal changes in RPE-disease areas. These areas were determined by pathological Hyperfluorescent areas using FA images between the various time points or over a long-term clinical course. Our program allowed us to reduce the workload of researchers and obtain a reproducible evaluation of the differences in fluorescence fundus images. Our program will contribute to the evaluation of RPE cell function and follow-up observations after iPSC-RPE transplantation.

## Supplementary Information

Supplementary Information.

## Data Availability

The data that support the findings of this study are available from the corresponding author, M.M., upon reasonable request.
