# Supplementary material for: Automated evaluation of retinal pigment epithelium disease area in eyes with age-related macular degeneration
Source: Sci Rep. 2022 Jan 18;12:892. doi: 10.1038/s41598-022-05006-3 (PMC8766591; doi:10.1038/s41598-022-05006-3)
Supplement: Supplementary file 1 — Supplementary Information. [file 41598_2022_5006_MOESM1_ESM.pdf]

Supplementary Information

Automated Evaluation of Retinal Pigment Epithelium Disease Area in Eyes with Age-related Macular Degeneration

Naohiro Motozawa[1,2,6], Takuya Miura[3,6], Koji Ochiai[3], Midori Yamamoto[1], Takaaki Horinouchi[3], Taku Tsuzuki[4], Genki N. Kanda[1,3], Yosuke Ozawa[4], Akitaka Tsujikawa[2], Koichi Takahashi[3], Masayo Takahashi[1,5], Yasuo Kurimoto[1], Tadao Maeda[1], Michiko Mandai[1,*]

[1] Kobe City Eye Hospital, 2-1-8 Minatojima Minamimachi, Chuo-ku, Kobe, Hyogo, 650-0047, Japana

[2] Department of Ophthalmology and Visual Sciences, Kyoto University Graduate School of Medicine, 54 Shogoin Kawahara-cho, Sakyo-ku, Kyoto, 606-8507, Japan

[3] Laboratory for Biologically Inspired Computing, RIKEN Center for Biosystems Dynamics Research. 6-2-3 Furuedai, Suita, Osaka, 565-0874, Japan

[4] Epistra Inc. 2-2-15 Hamamatsu-cho, Minato-ku, Tokyo, 105-0013, Japan

[5] Vision Care Cell Therapy Inc. Kobe Eye Center 5F, 2-1-8 Minatojima-minamimachi, Chuo-ku, Kobe, Hyogo, 650-0047, Japan

[6] First authors (equal contribution)

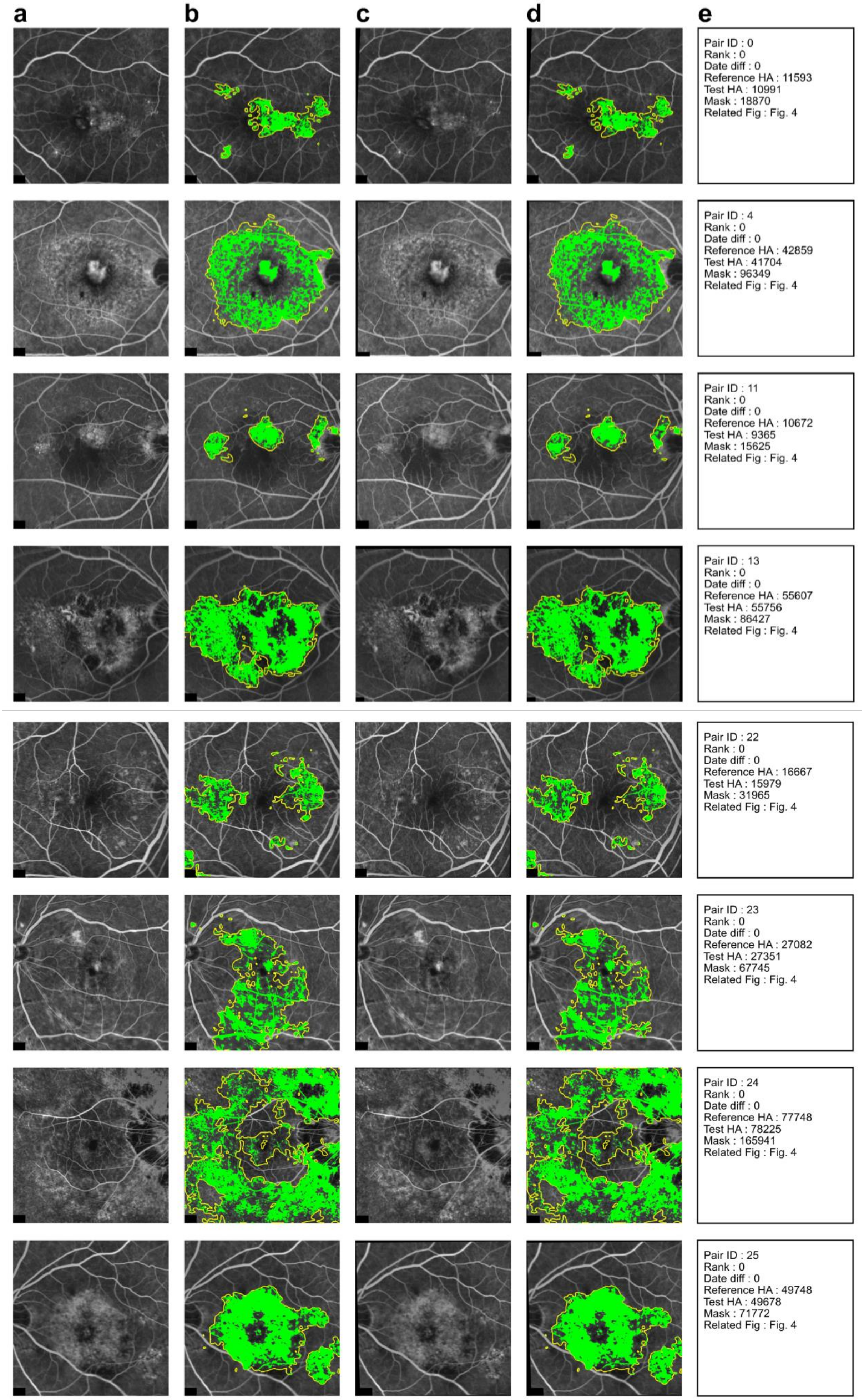
a
b
c
d
e
Pair ID : 0
Rank : 0
Date diff : 0
Reference HA : 11593
Test HA : 10991
Mask : 18870
Related Fig : Fig. 4
Pair ID : 4
Rank : 0
Date diff : 0
Reference HA : 42859
Test HA : 41704
Mask : 96349
Related Fig : Fig. 4
Pair ID : 11
Rank : 0
Date diff : 0
Reference HA : 10672
Test HA : 9365
Mask : 15625
Related Fig : Fig. 4
Pair ID : 13
Rank : 0
Date diff : 0
Reference HA : 55607
Test HA : 55756
Mask : 86427
Related Fig : Fig. 4
Pair ID : 22
Rank : 0
Date diff : 0
Reference HA : 16667
Test HA : 15979
Mask : 31965
Related Fig : Fig. 4
Pair ID : 23
Rank : 0
Date diff : 0
Reference HA : 27082
Test HA : 27351
Mask : 67745
Related Fig : Fig. 4
Pair ID : 24
Rank : 0
Date diff : 0
Reference HA : 77748
Test HA : 78225
Mask : 165941
Related Fig : Fig. 4
Pair ID : 25
Rank : 0
Date diff : 0
Reference HA : 49748
Test HA : 49678
Mask : 71772
Related Fig : Fig. 4

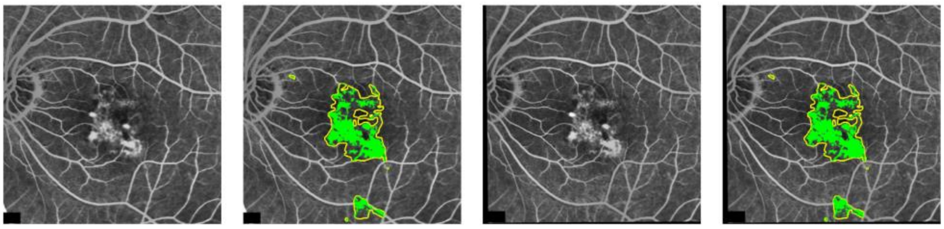

Pair ID : 26
Rank : 0
Date diff : 0
Reference HA : 8530
Test HA : 9088
Mask : 17512
Related Fig : Fig. 4

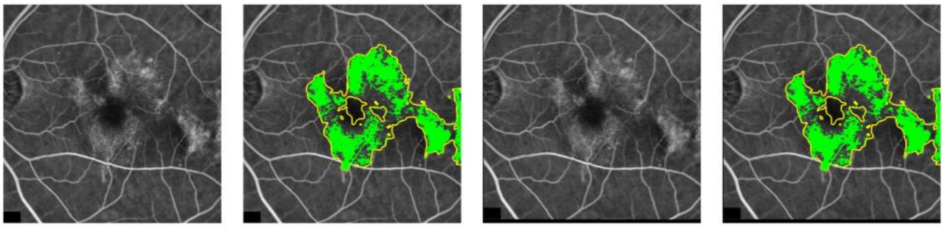

Pair ID : 27
Rank : 0
Date diff : 0
Reference HA : 23934
Test HA : 24601
Mask : 44916
Related Fig : Fig. 4

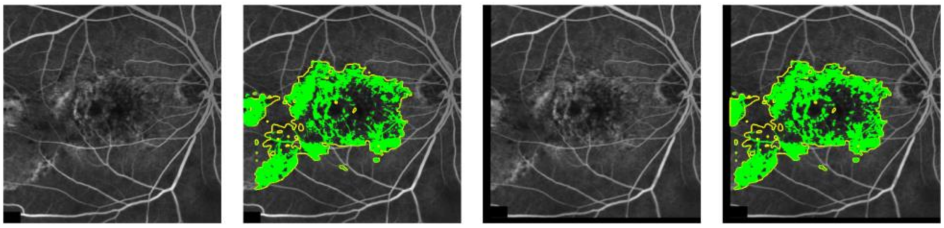

Pair ID : 28
Rank : 0
Date diff : 0
Reference HA : 29080
Test HA : 29843
Mask : 55916
Related Fig : Fig. 4

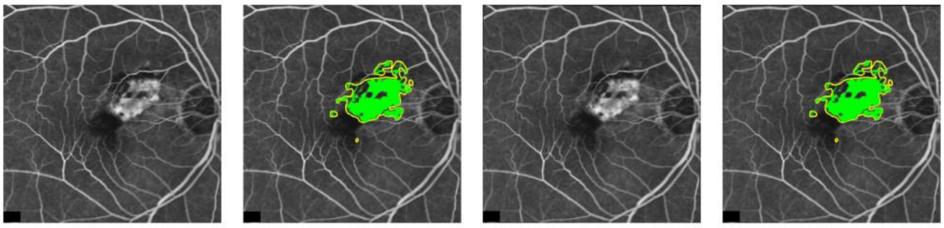

Pair ID : 29
Rank : 0
Date diff : 0
Reference HA : 8469
Test HA : 8657
Mask : 12711
Related Fig : Fig. 4

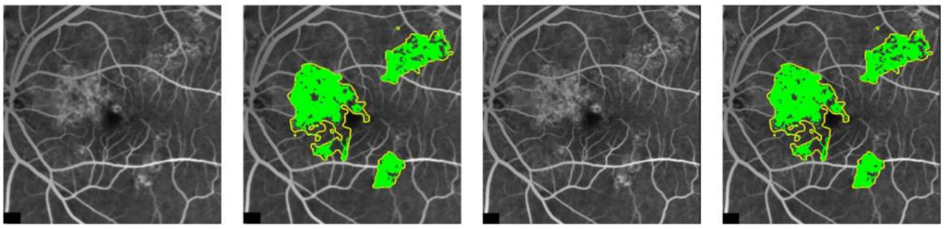

Pair ID : 30
Rank : 0
Date diff : 0
Reference HA : 24780
Test HA : 24621
Mask : 34441
Related Fig : Fig. 4

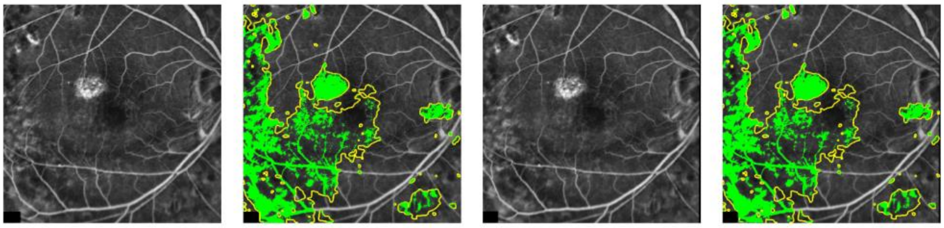

Pair ID : 31
Rank : 0
Date diff : 0
Reference HA : 34501
Test HA : 34653
Mask : 83035
Related Fig : Fig. 4

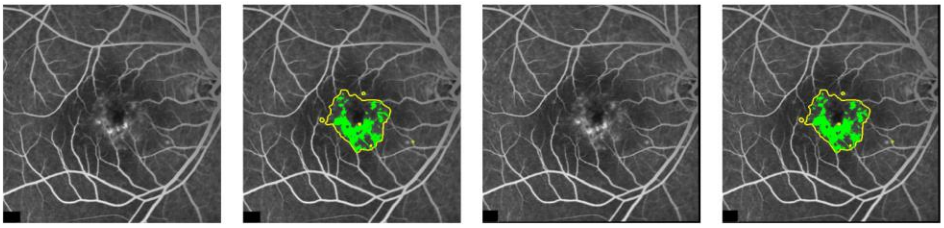

Pair ID : 32
Rank : 0
Date diff : 0
Reference HA : 4352
Test HA : 4892
Mask : 12966
Related Fig : Fig. 4

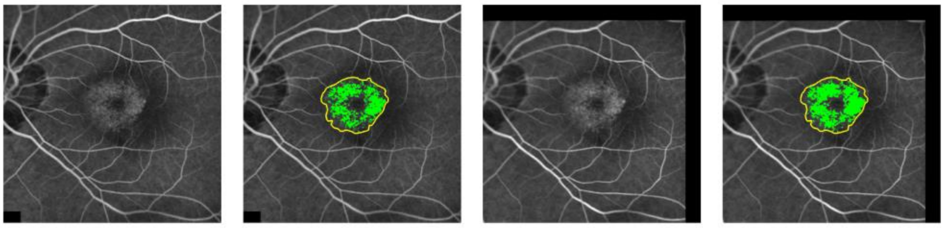

Pair ID : 33
Rank : 0
Date diff : 0
Reference HA : 5194
Test HA : 6726
Mask : 13860
Related Fig : Fig. 4

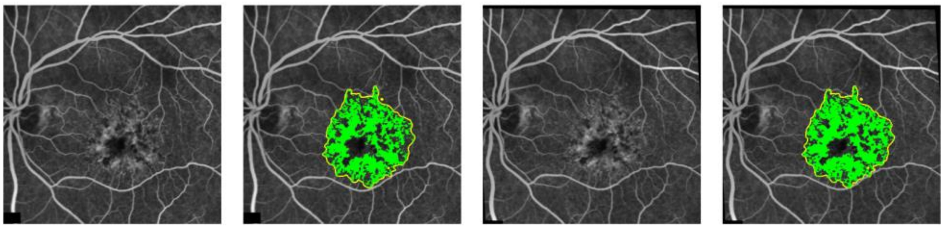

Pair ID : 34
Rank : 0
Date diff : 0
Reference HA : 16697
Test HA : 18716
Mask : 30463
Related Fig : Fig. 4

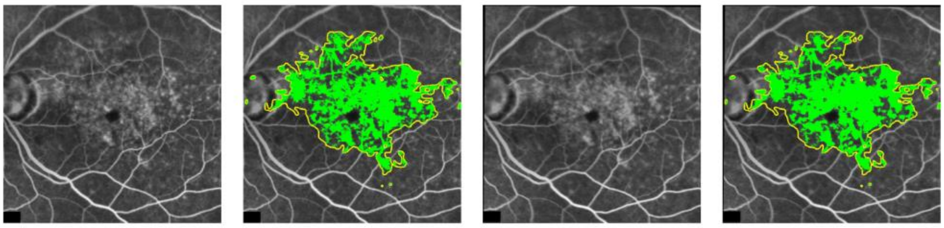

Pair ID : 35
Rank : 0
Date diff : 0
Reference HA : 35412
Test HA : 36390
Mask : 60546
Related Fig : Fig. 4

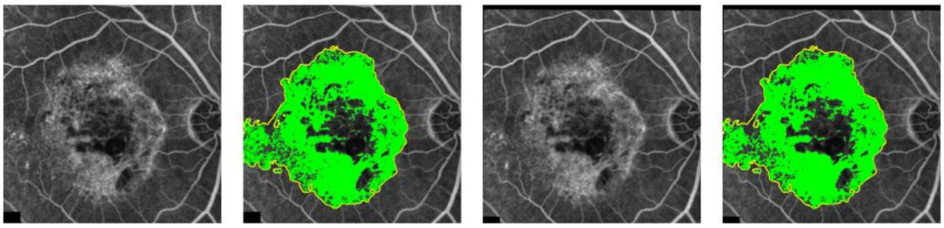

Pair ID : 36
Rank : 0
Date diff : 0
Reference HA : 57940
Test HA : 58760
Mask : 86172
Related Fig : Fig. 4

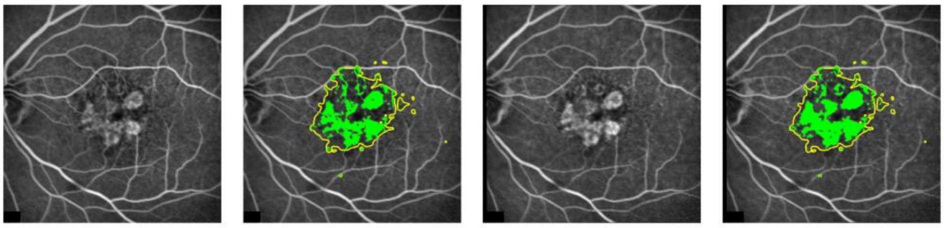

Pair ID : 37
Rank : 0
Date diff : 0
Reference HA : 9634
Test HA : 11056
Mask : 25322
Related Fig : Fig. 4

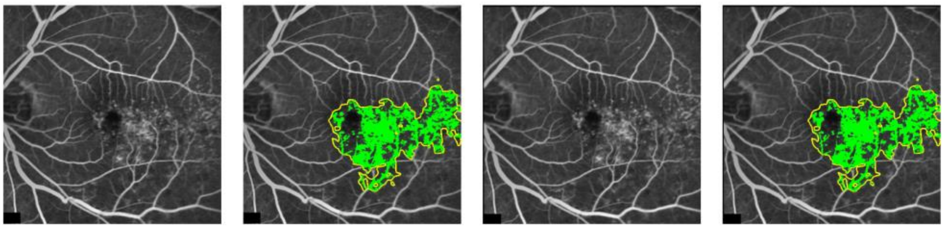

Pair ID : 38
Rank : 0
Date diff : 0
Reference HA : 19280
Test HA : 19568
Mask : 36187
Related Fig : Fig. 4

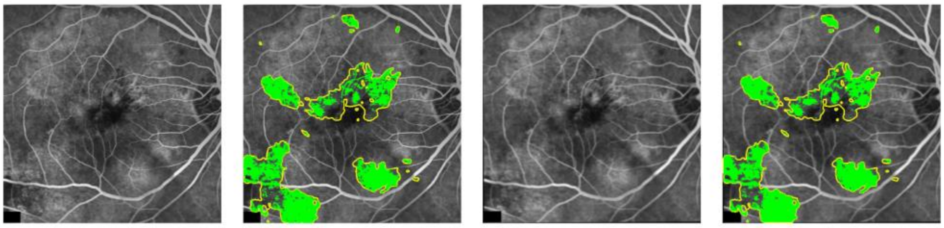

Pair ID : 39
Rank : 0
Date diff : 0
Reference HA : 24374
Test HA : 24859
Mask : 39039
Related Fig : Fig. 4

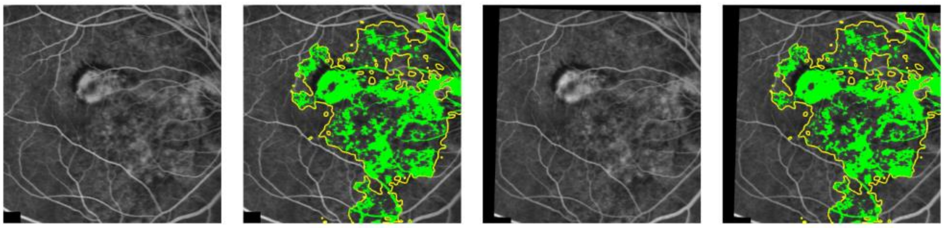

Pair ID : 40
Rank : 0
Date diff : 0
Reference HA : 38942
Test HA : 39167
Mask : 97059
Related Fig : Fig. 4

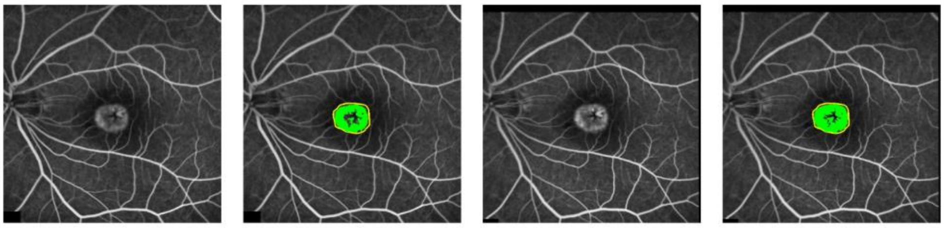

Pair ID : 41
Rank : 0
Date diff : 0
Reference HA : 3151
Test HA : 3596
Mask : 4649
Related Fig : Fig. 4

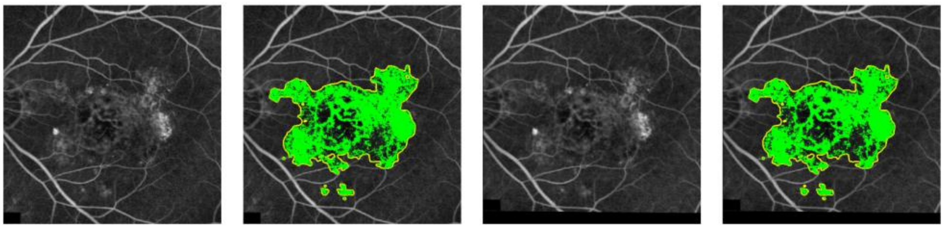

Pair ID : 42
Rank : 0
Date diff : 0
Reference HA : 33910
Test HA : 33304
Mask : 51099
Related Fig : Fig. 4

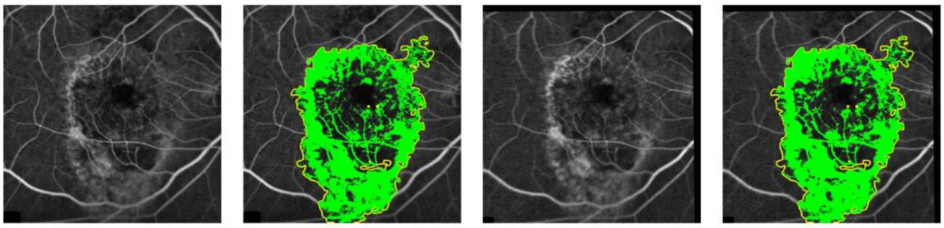

Pair ID : 43
Rank : 0
Date diff : 0
Reference HA : 50580
Test HA : 53090
Mask : 89041
Related Fig : Fig. 4

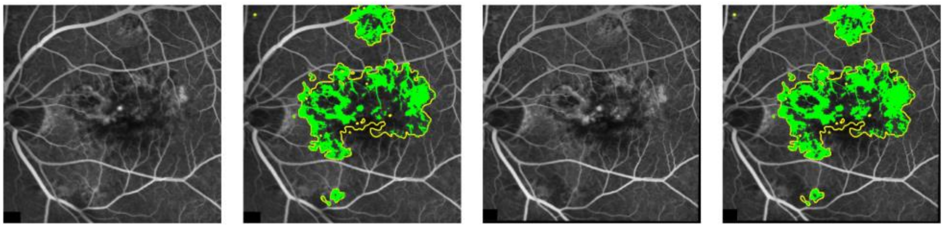

Pair ID : 44
Rank : 0
Date diff : 0
Reference HA : 21246
Test HA : 25773
Mask : 48827
Related Fig : Fig. 4

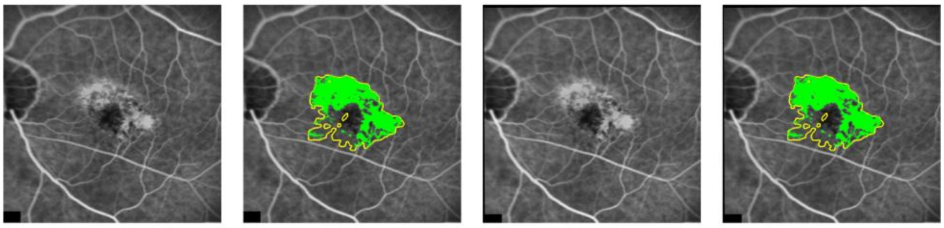

Pair ID : 46
Rank : 0
Date diff : 0
Reference HA : 12464
Test HA : 12682
Mask : 22698
Related Fig : Fig. 4

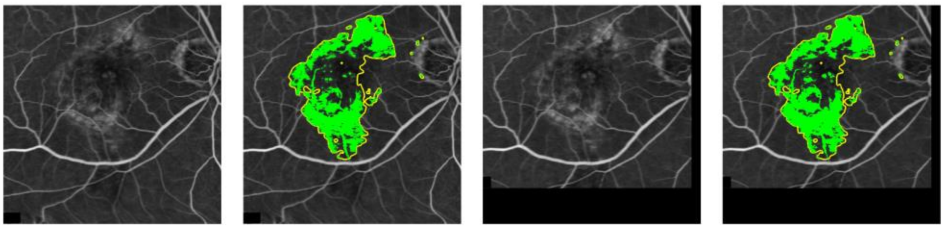

Pair ID : 47
Rank : 0
Date diff : 0
Reference HA : 20998
Test HA : 22888
Mask : 38823
Related Fig : Fig. 4

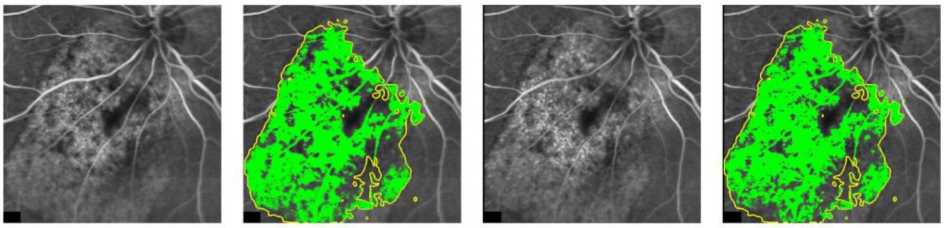

Pair ID : 48
Rank : 0
Date diff : 0
Reference HA : 67967
Test HA : 69023
Mask : 114447
Related Fig : Fig. 4

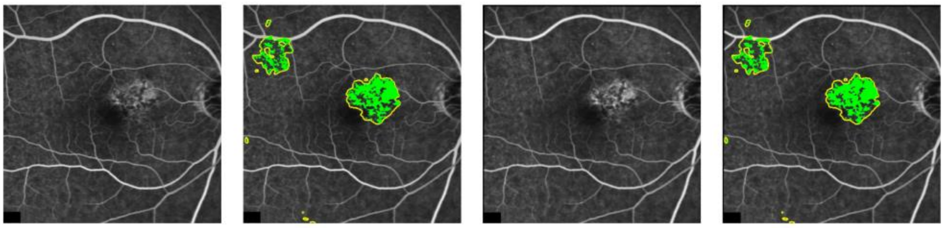

Pair ID : 49
Rank : 0
Date diff : 0
Reference HA : 7436
Test HA : 7829
Mask : 13828
Related Fig : Fig. 4

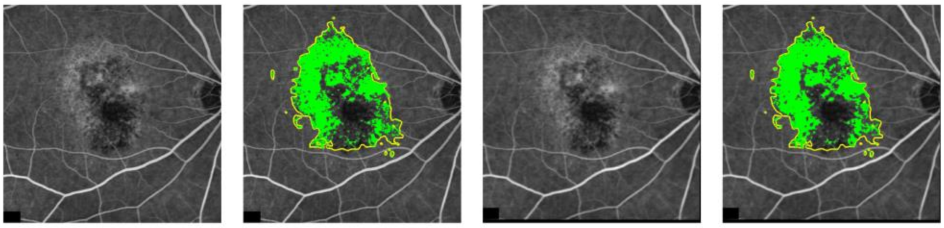

Pair ID : 50
Rank : 0
Date diff : 0
Reference HA : 22947
Test HA : 24175
Mask : 45670
Related Fig : Fig. 4

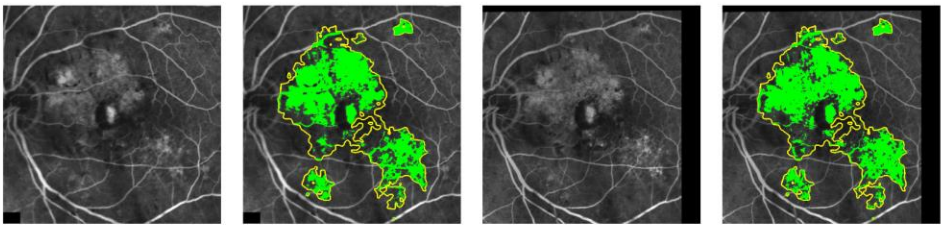

Pair ID : 59
Rank : 0
Date diff : 0
Reference HA : 31774
Test HA : 33568
Mask : 63792
Related Fig : Fig. 4

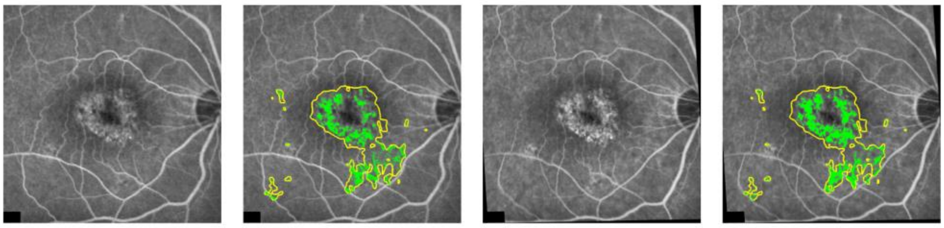

Pair ID : 62
Rank : 0
Date diff : 0
Reference HA : 6073
Test HA : 7845
Mask : 25405
Related Fig : Fig. 4

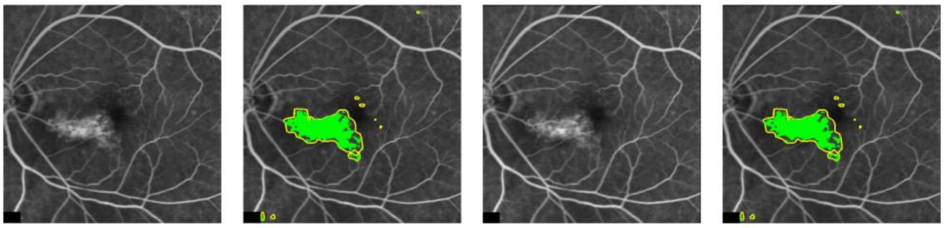

Pair ID : 63
Rank : 0
Date diff : 0
Reference HA : 7696
Test HA : 7431
Mask : 10970
Related Fig : Fig. 4

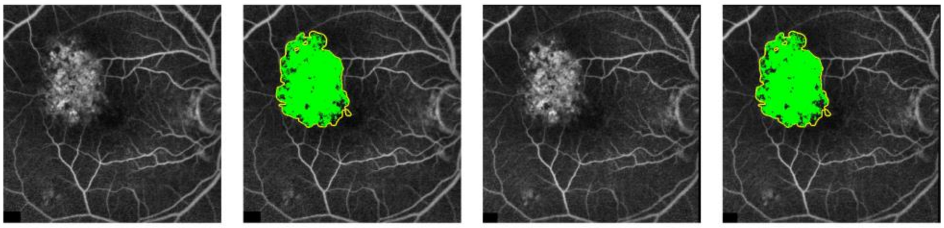

Pair ID : 64
Rank : 0
Date diff : 0
Reference HA : 21388
Test HA : 21238
Mask : 25530
Related Fig : Fig. 4

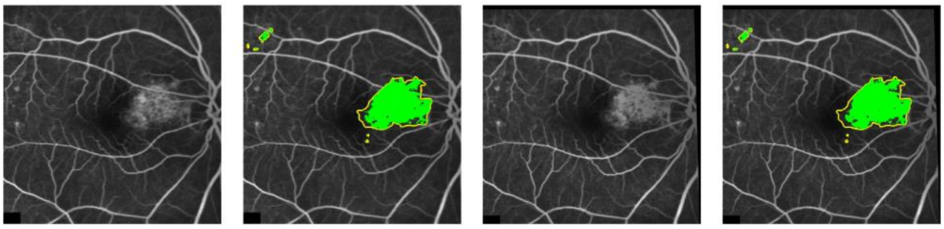

Pair ID : 65
Rank : 0
Date diff : 0
Reference HA : 11081
Test HA : 10868
Mask : 13652
Related Fig : Fig. 4

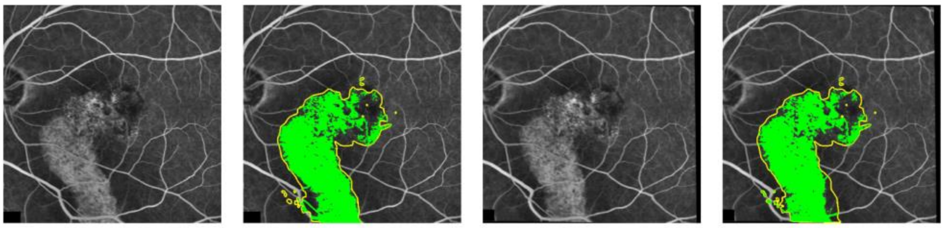

Pair ID : 66
Rank : 0
Date diff : 0
Reference HA : 31903
Test HA : 31760
Mask : 46184
Related Fig : Fig. 4

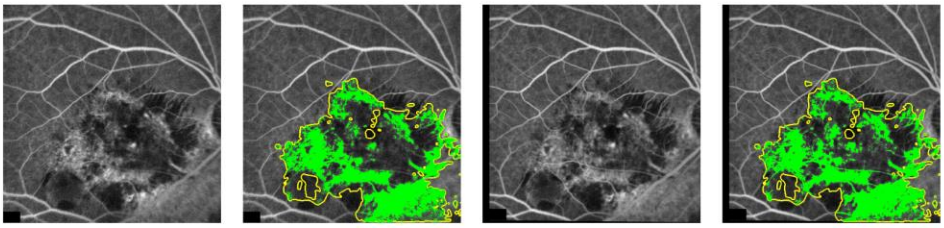

Pair ID : 67
Rank : 0
Date diff : 0
Reference HA : 34155
Test HA : 34805
Mask : 80672
Related Fig : Fig. 4

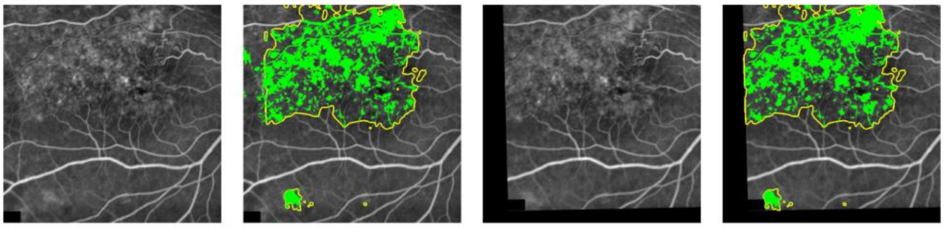

Pair ID : 68
Rank : 0
Date diff : 0
Reference HA : 32230
Test HA : 31625
Mask : 84916
Related Fig : Fig. 4

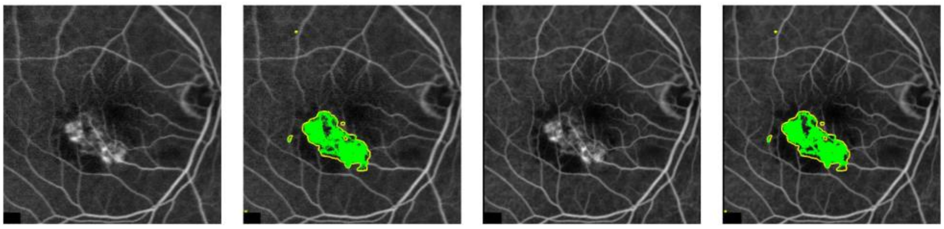

Pair ID : 69
Rank : 0
Date diff : 0
Reference HA : 7970
Test HA : 7784
Mask : 10586
Related Fig : Fig. 4

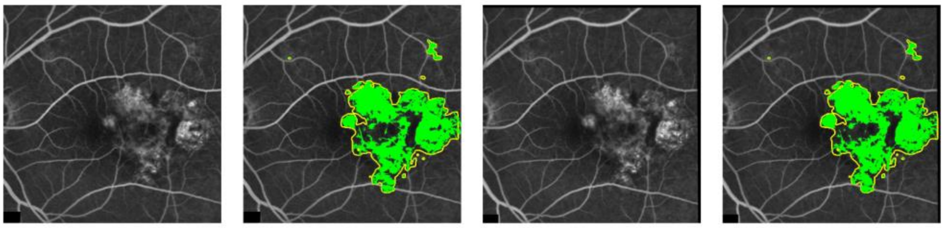

Pair ID : 70
Rank : 0
Date diff : 0
Reference HA : 24536
Test HA : 25035
Mask : 38288
Related Fig : Fig. 4

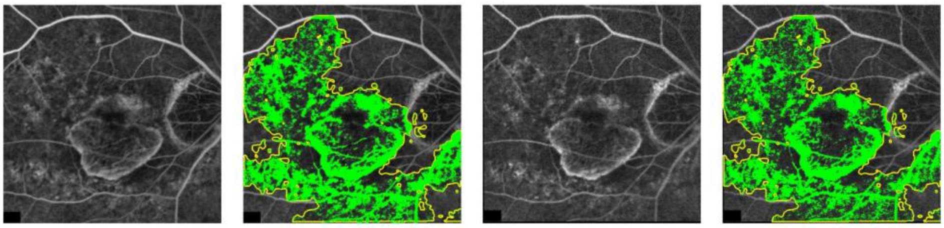

Pair ID : 71
Rank : 0
Date diff : 0
Reference HA : 60533
Test HA : 56211
Mask : 130672
Related Fig : Fig. 4

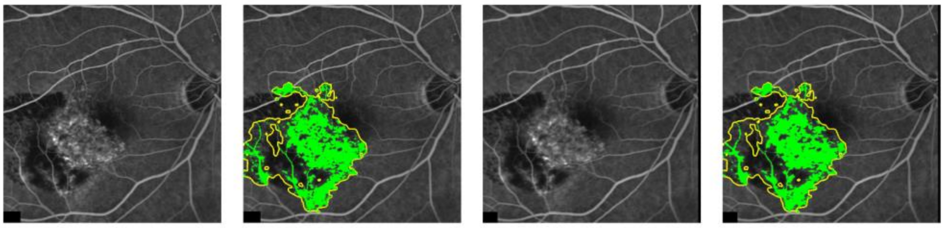

Pair ID : 72
Rank : 0
Date diff : 0
Reference HA : 20959
Test HA : 20803
Mask : 44021
Related Fig : Fig. 4

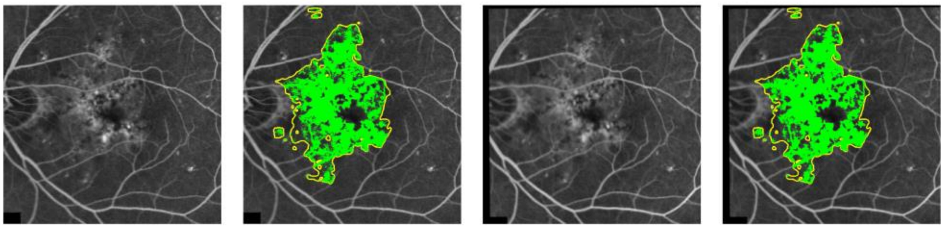

Pair ID : 73
Rank : 0
Date diff : 0
Reference HA : 31231
Test HA : 32160
Mask : 50111
Related Fig : Fig. 4

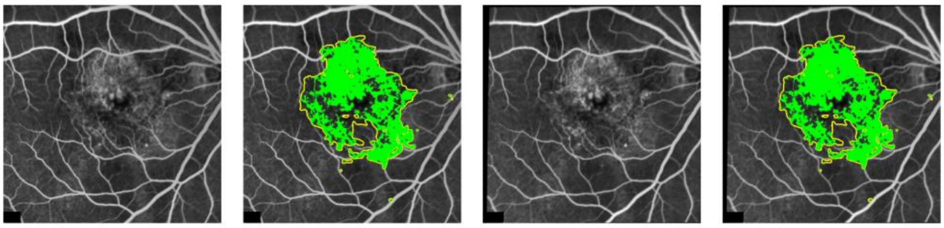

Pair ID : 74
Rank : 0
Date diff : 0
Reference HA : 29566
Test HA : 30866
Mask : 44807
Related Fig : Fig. 4

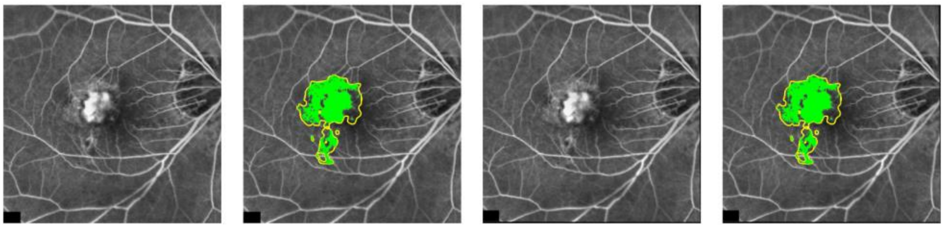

Pair ID : 75
Rank : 0
Date diff : 0
Reference HA : 9363
Test HA : 9435
Mask : 14370
Related Fig : Fig. 4

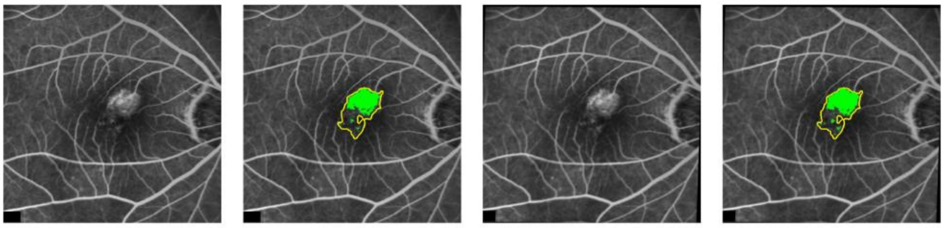

Pair ID : 76
Rank : 0
Date diff : 0
Reference HA : 2776
Test HA : 2955
Mask : 6250
Related Fig : Fig. 4

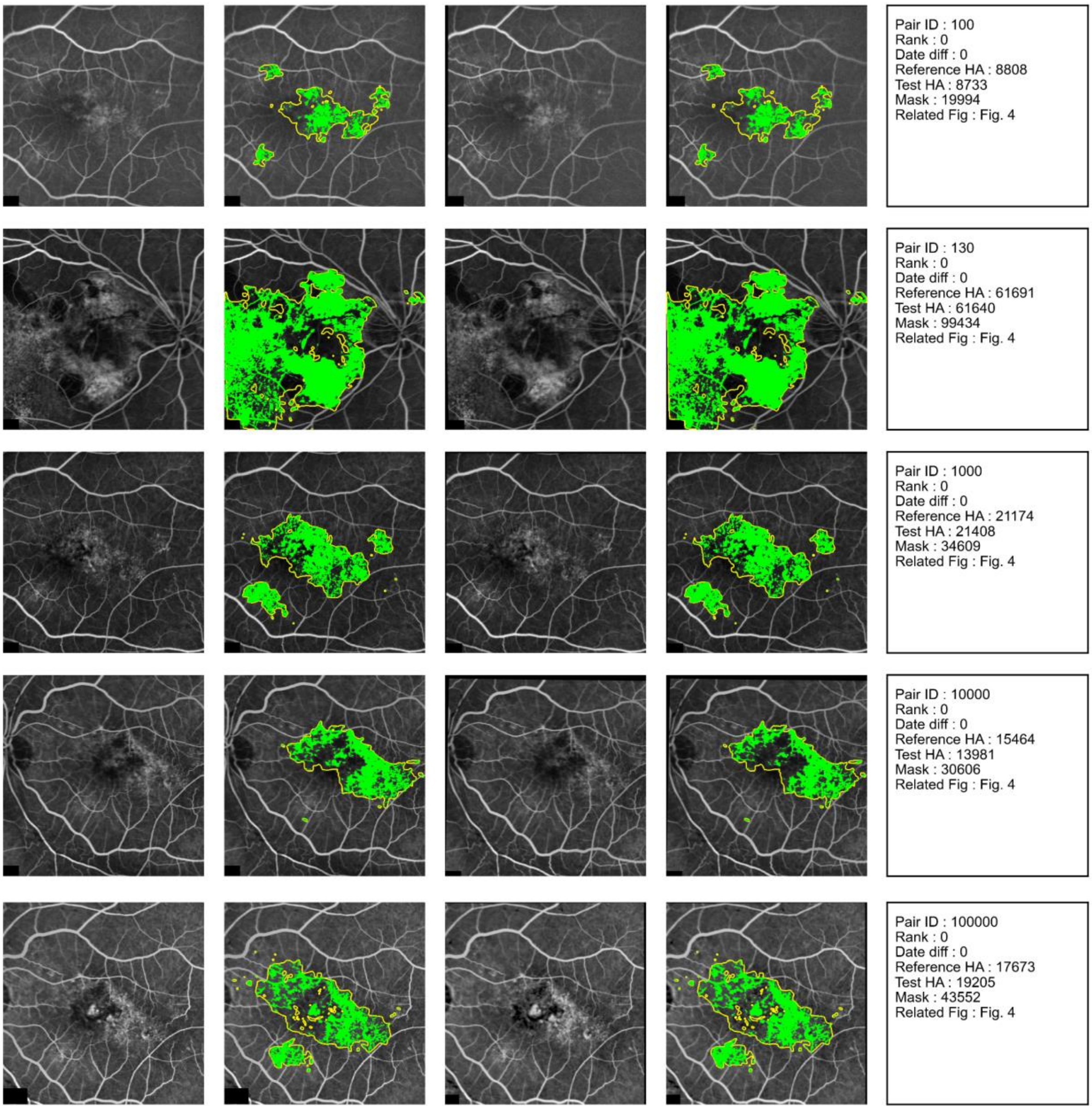

Figure S1. All pairs of images taken on the same day

All the 53 pairs of images taken on the same day (28 patients).

(**a**), (**c**) A pair of aligned FA images, brightness-corrected by image processing.

(**b**), (**d**) A pair of fundus FA images with Predicted Abnormal regions (yellow) and Hyperfluorescent areas (green) after binarization.

(**e**) "Pair ID" is a unique ID for each pair. "Rank 0" means image pairs taken on the same day. "Date diff" is the difference between the acquisition dates of the two images, and "0" means that the images were taken on the same day. "Reference HA" is the Hyperfluorescent area output by (**a**) and (**b**), and "Test HA" is the Hyperfluorescent area output by (**c**) and (**d**). "Mask" is the area of the Abnormal region. "Related Fig." refers to the related one in the main figure.

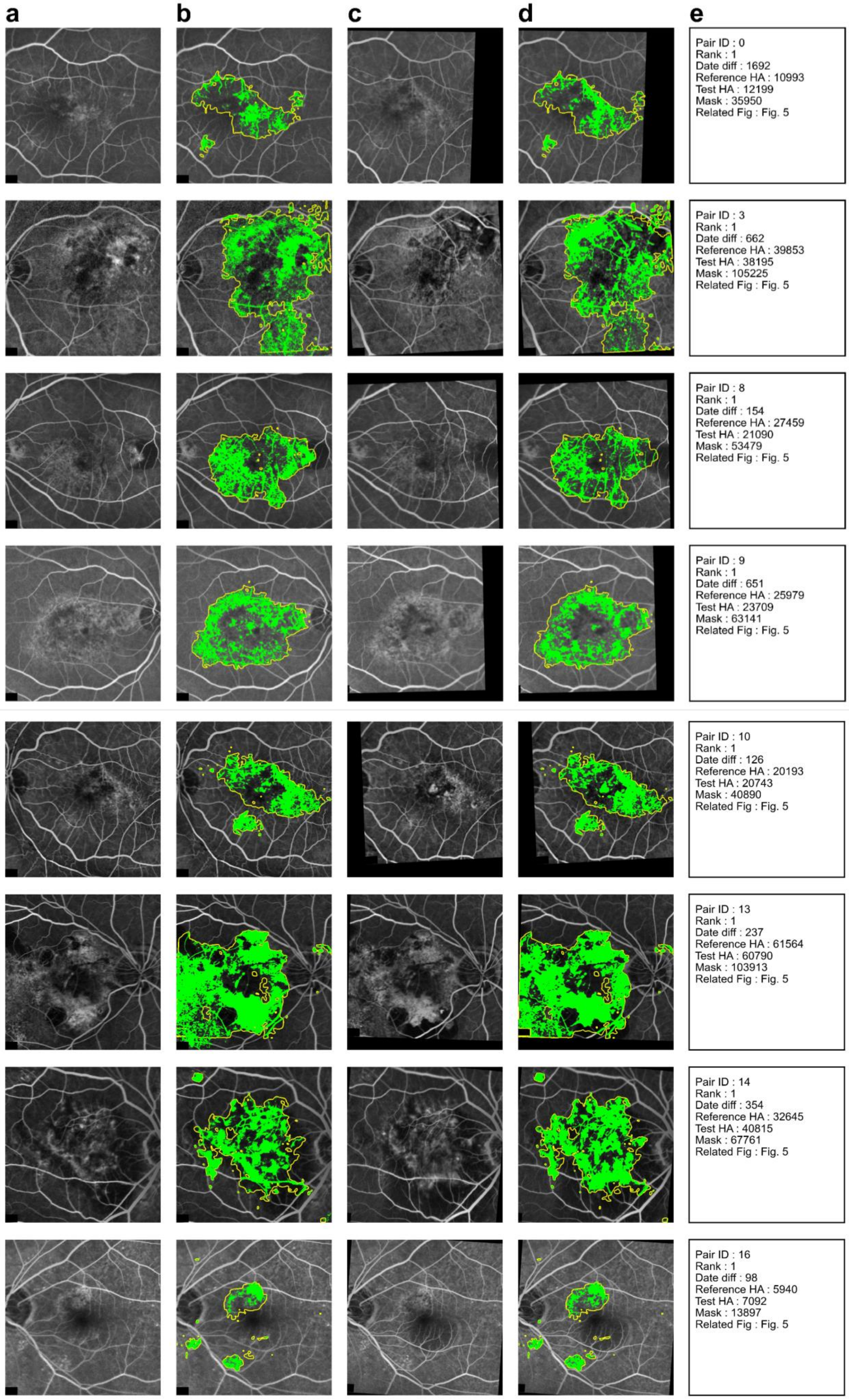
a
b
c
d
e
Pair ID : 0
Rank : 1
Date diff : 1692
Reference HA : 10993
Test HA : 12199
Mask : 35950
Related Fig : Fig. 5
Pair ID : 3
Rank : 1
Date diff : 662
Reference HA : 39853
Test HA : 38195
Mask : 105225
Related Fig : Fig. 5
Pair ID : 8
Rank : 1
Date diff : 154
Reference HA : 27459
Test HA : 21090
Mask : 53479
Related Fig : Fig. 5
Pair ID : 9
Rank : 1
Date diff : 651
Reference HA : 25979
Test HA : 23709
Mask : 63141
Related Fig : Fig. 5
Pair ID : 10
Rank : 1
Date diff : 126
Reference HA : 20193
Test HA : 20743
Mask : 40890
Related Fig : Fig. 5
Pair ID : 13
Rank : 1
Date diff : 237
Reference HA : 61564
Test HA : 60790
Mask : 103913
Related Fig : Fig. 5
Pair ID : 14
Rank : 1
Date diff : 354
Reference HA : 32645
Test HA : 40815
Mask : 67761
Related Fig : Fig. 5
Pair ID : 16
Rank : 1
Date diff : 98
Reference HA : 5940
Test HA : 7092
Mask : 13897
Related Fig : Fig. 5

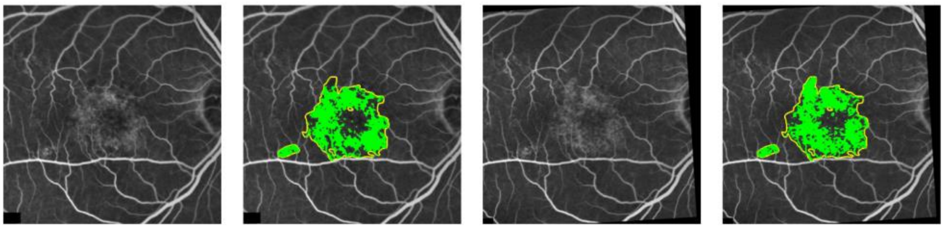

Pair ID : 19
Rank : 1
Date diff : 581
Reference HA : 15365
Test HA : 15942
Mask : 25118
Related Fig : Fig. 5

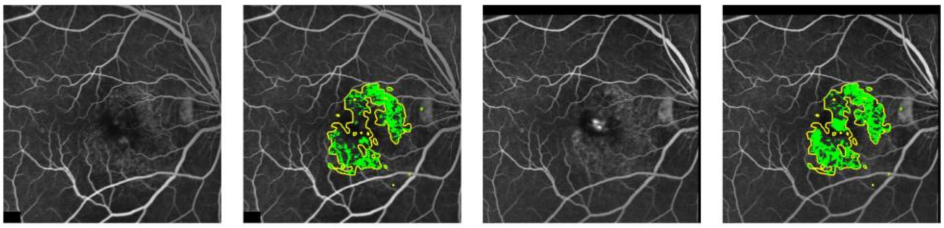

Pair ID : 20
Rank : 1
Date diff : 115
Reference HA : 6883
Test HA : 9147
Mask : 18478
Related Fig : Fig. 5

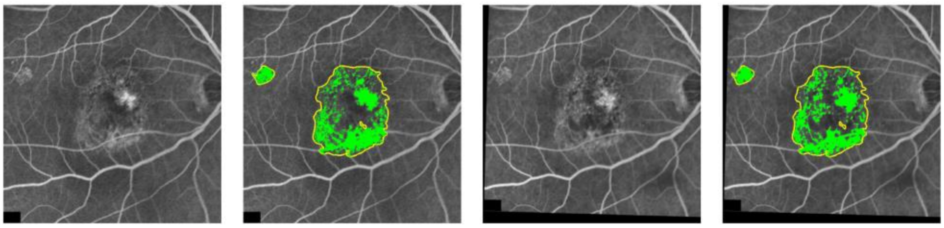

Pair ID : 21
Rank : 1
Date diff : 98
Reference HA : 11571
Test HA : 11514
Mask : 29135
Related Fig : Fig. 5

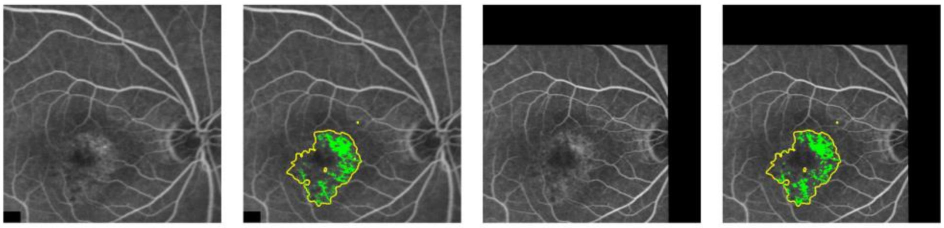

Pair ID : 45
Rank : 1
Date diff : 226
Reference HA : 3457
Test HA : 3750
Mask : 16725
Related Fig : Fig. 5

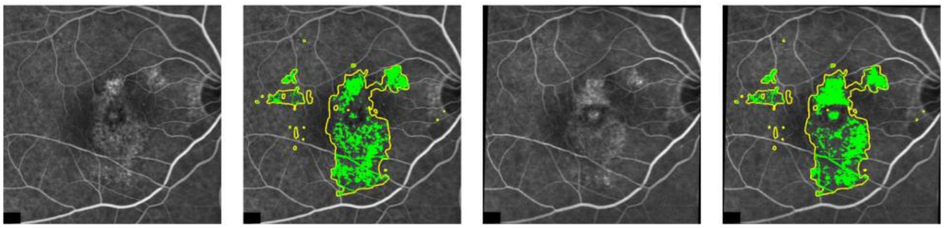

Pair ID : 60
Rank : 1
Date diff : 2454
Reference HA : 12370
Test HA : 13634
Mask : 33538
Related Fig : Fig. 5

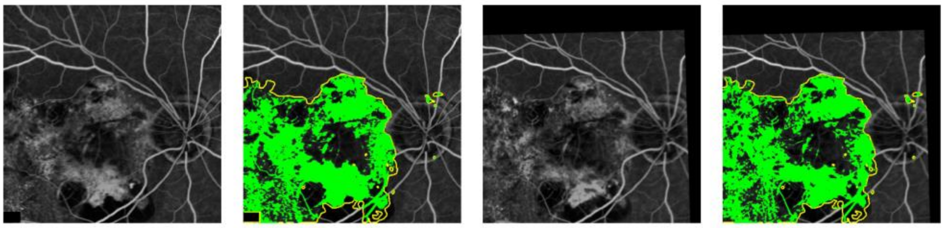

Pair ID : 130
Rank : 1
Date diff : 44
Reference HA : 54489
Test HA : 52920
Mask : 97366
Related Fig : Fig. 5

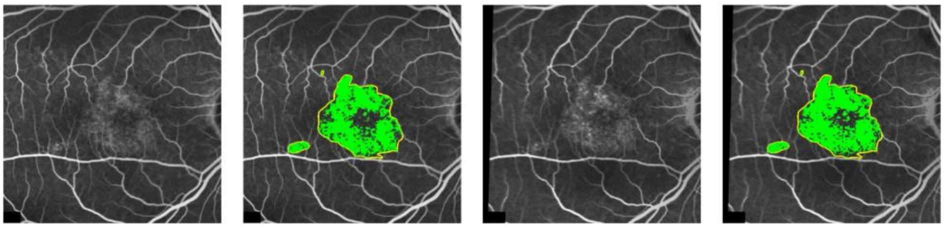

Pair ID : 190
Rank : 1
Date diff : 91
Reference HA : 16561
Test HA : 17853
Mask : 24317
Related Fig : Fig. 5

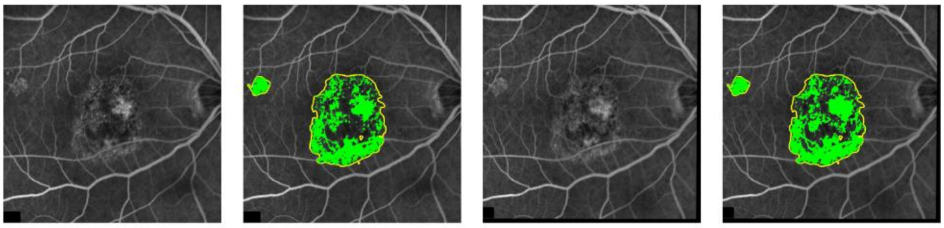

Pair ID : 210
Rank : 1
Date diff : 93
Reference HA : 14153
Test HA : 15754
Mask : 29851
Related Fig : Fig. 5

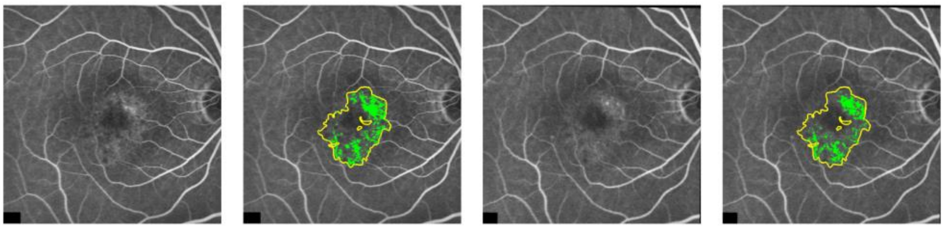

Pair ID : 450
Rank : 1
Date diff : 759
Reference HA : 3770
Test HA : 3086
Mask : 16840
Related Fig : Fig. 5

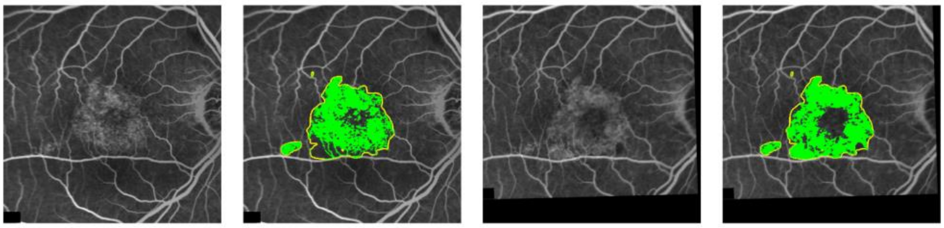

Pair ID : 1900
Rank : 1
Date diff : 918
Reference HA : 17941
Test HA : 18790
Mask : 26971
Related Fig : Fig. 5

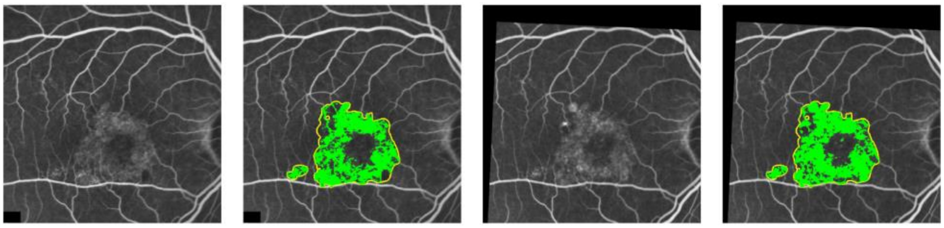

Pair ID : 19000
Rank : 1
Date diff : 231
Reference HA : 16691
Test HA : 18372
Mask : 28910
Related Fig : Fig. 5

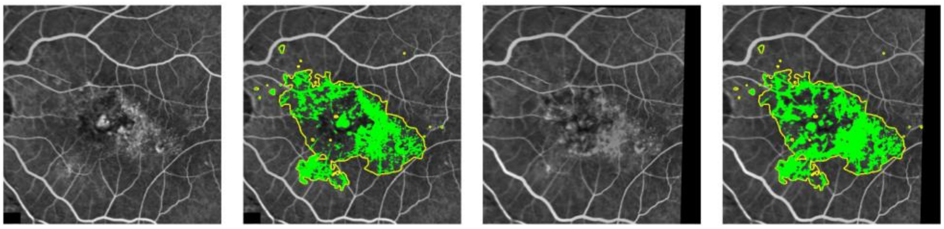

Pair ID : 0
Rank : 2
Date diff : 359
Reference HA : 21353
Test HA : 25723
Mask : 49456
Related Fig : Fig. 5

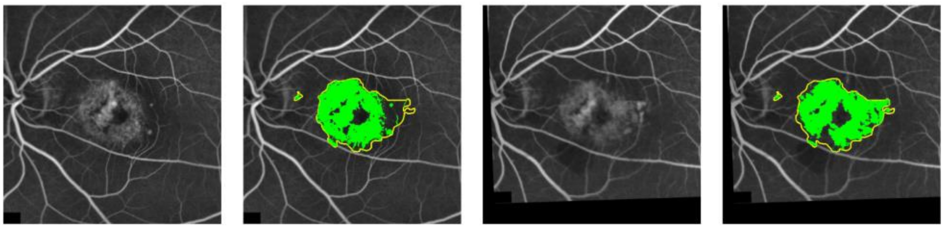

Pair ID : 1
Rank : 2
Date diff : 1036
Reference HA : 15513
Test HA : 16477
Mask : 22907
Related Fig : Fig. 5

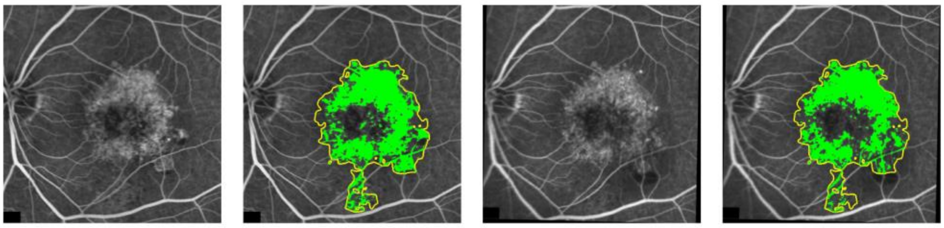

Pair ID : 5
Rank : 2
Date diff : 313
Reference HA : 26487
Test HA : 24913
Mask : 50325
Related Fig : Fig. 5

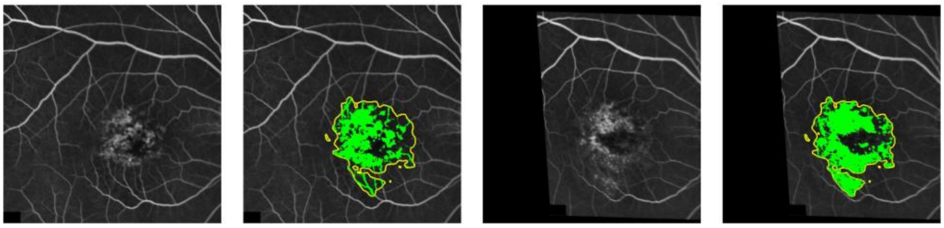

Pair ID : 6
Rank : 2
Date diff : 715
Reference HA : 11939
Test HA : 15652
Mask : 25704
Related Fig : Fig. 5

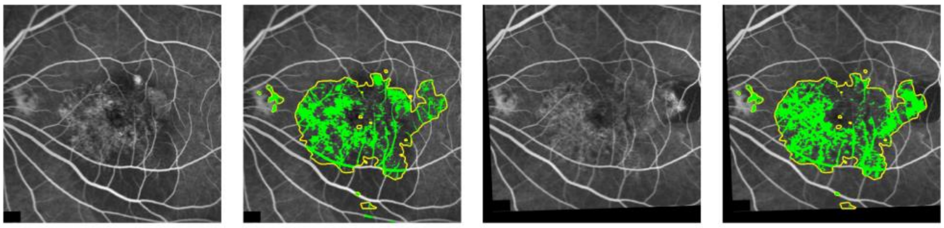

Pair ID : 8
Rank : 2
Date diff : 406
Reference HA : 17933
Test HA : 22541
Mask : 52790
Related Fig : Fig. 5

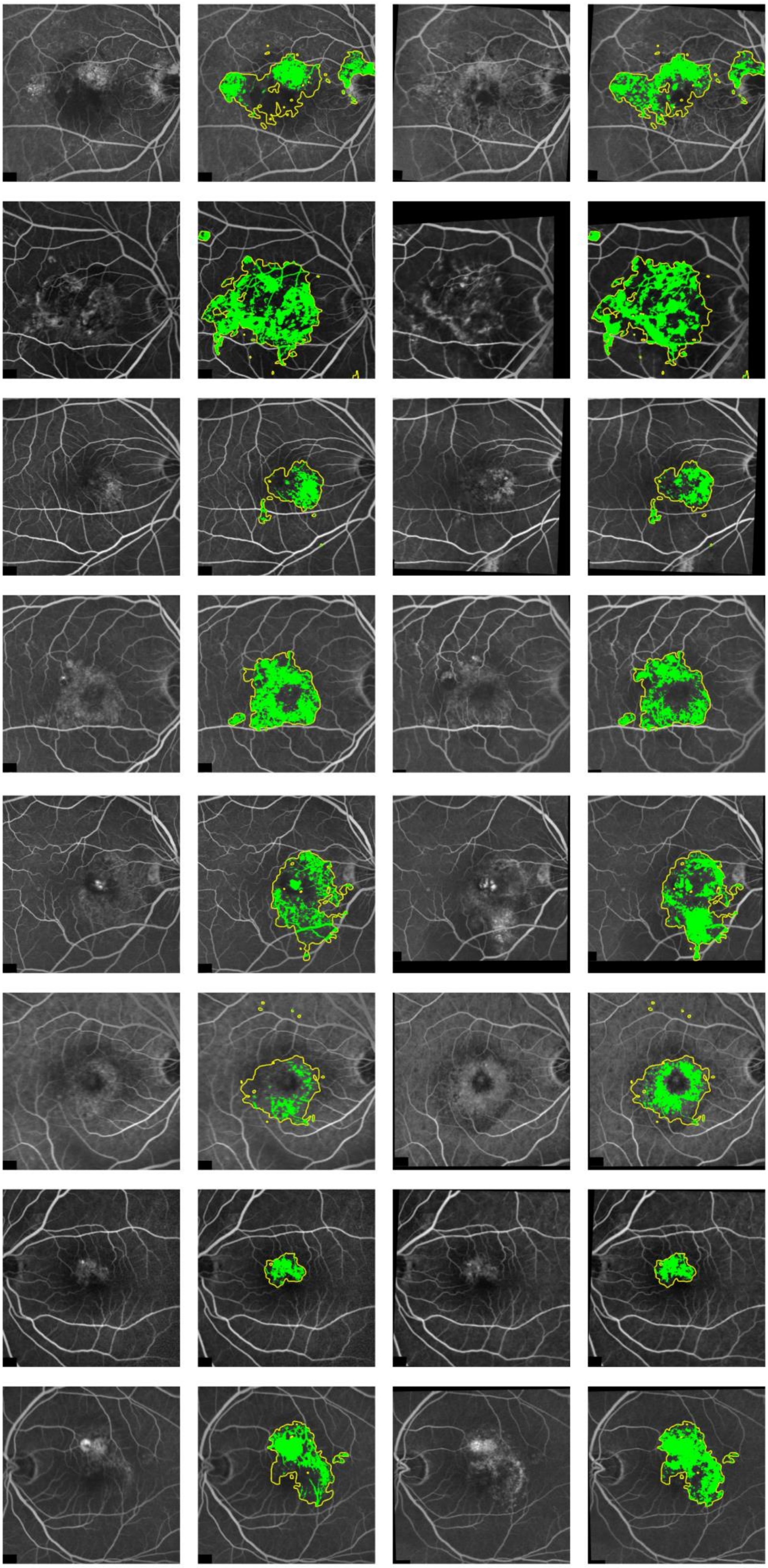

Pair ID : 11
Rank : 2
Date diff : 329
Reference HA : 12544
Test HA : 16138
Mask : 34916
Related Fig : Fig. 5

Pair ID : 14
Rank : 2
Date diff : 565
Reference HA : 29578
Test HA : 27876
Mask : 55963
Related Fig : Fig. 5

Pair ID : 17
Rank : 2
Date diff : 3209
Reference HA : 5720
Test HA : 5997
Mask : 15351
Related Fig : Fig. 5

Pair ID : 19
Rank : 2
Date diff : 213
Reference HA : 19393
Test HA : 14904
Mask : 33338
Related Fig : Fig. 5

Pair ID : 20
Rank : 2
Date diff : 1386
Reference HA : 11385
Test HA : 15763
Mask : 35446
Related Fig : Fig. 5

Pair ID : 45
Rank : 2
Date diff : 1851
Reference HA : 4334
Test HA : 10180
Mask : 27990
Related Fig : Fig. 5

Pair ID : 51
Rank : 2
Date diff : 734
Reference HA : 3461
Test HA : 3880
Mask : 6961
Related Fig : Fig. 5

Pair ID : 52
Rank : 2
Date diff : 998
Reference HA : 11015
Test HA : 15929
Mask : 26121
Related Fig : Fig. 5

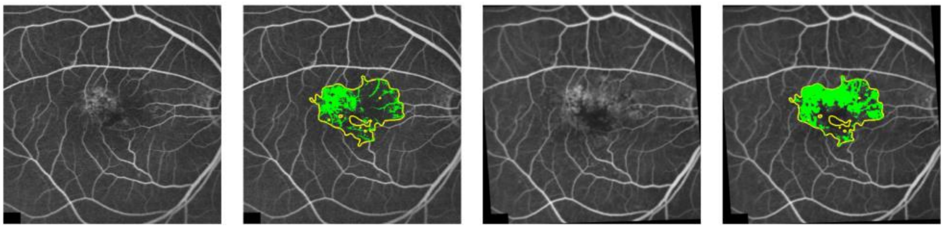

Pair ID : 53
Rank : 2
Date diff : 3474
Reference HA : 5466
Test HA : 9436
Mask : 17922
Related Fig : Fig. 5

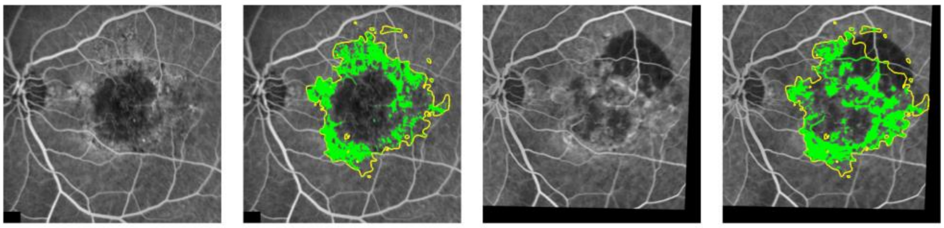

Pair ID : 54
Rank : 2
Date diff : 693
Reference HA : 21590
Test HA : 18583
Mask : 56289
Related Fig : Fig. 5

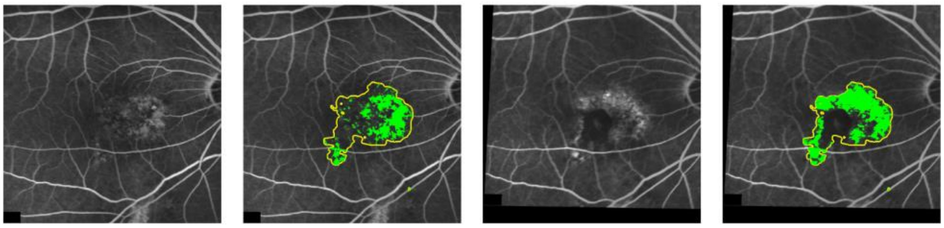

Pair ID : 170
Rank : 2
Date diff : 1262
Reference HA : 6394
Test HA : 10342
Mask : 21080
Related Fig : Fig. 5

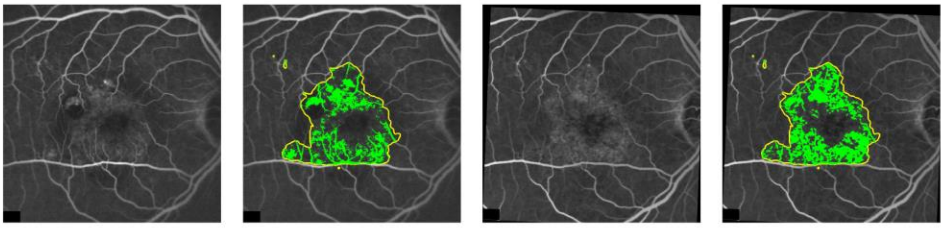

Pair ID : 190
Rank : 2
Date diff : 445
Reference HA : 11420
Test HA : 15858
Mask : 37171
Related Fig : Fig. 5

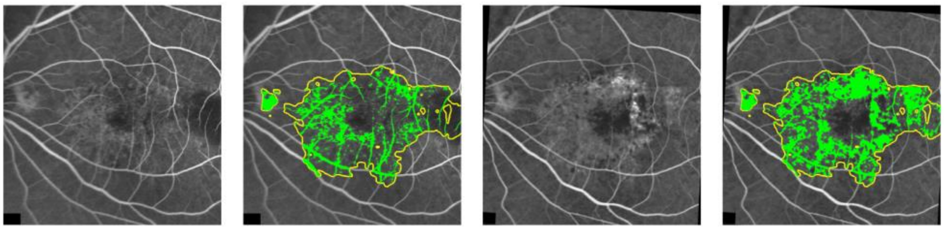

Pair ID : 800
Rank : 2
Date diff : 703
Reference HA : 16582
Test HA : 26886
Mask : 66358
Related Fig : Fig. 5

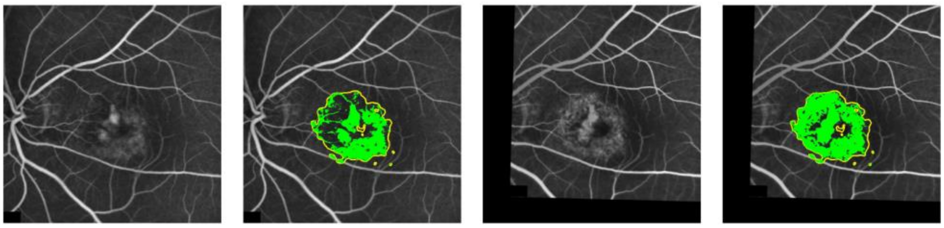

Pair ID : 1
Rank : 3
Date diff : 365
Reference HA : 10599
Test HA : 15267
Mask : 20433
Related Fig : Fig. 5

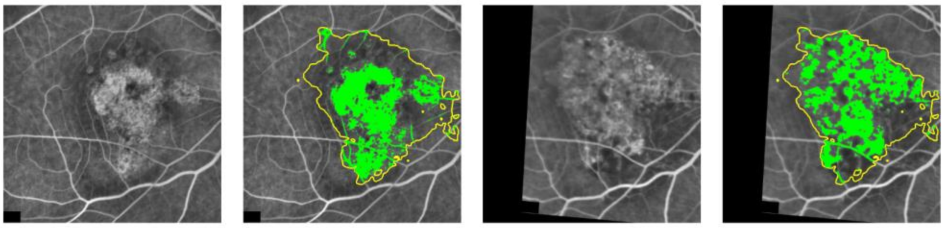

Pair ID : 2
Rank : 3
Date diff : 1349
Reference HA : 24934
Test HA : 28577
Mask : 70944
Related Fig : Fig. 5

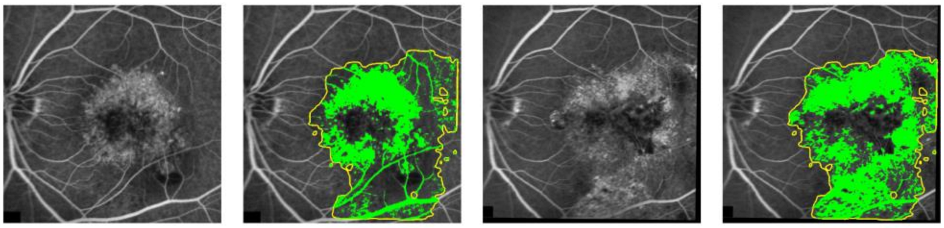

Pair ID : 5
Rank : 3
Date diff : 651
Reference HA : 35069
Test HA : 48835
Mask : 95298
Related Fig : Fig. 5

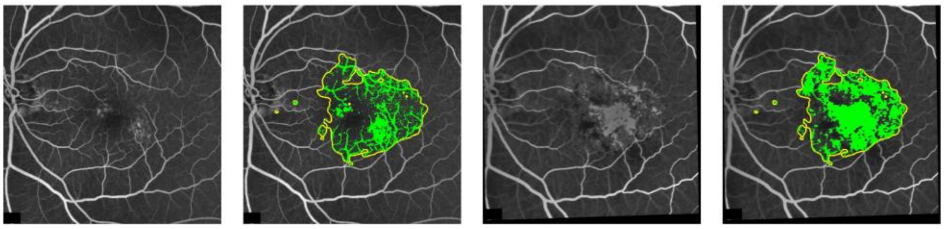

Pair ID : 7
Rank : 3
Date diff : 2128
Reference HA : 10742
Test HA : 20316
Mask : 36591
Related Fig : Fig. 5

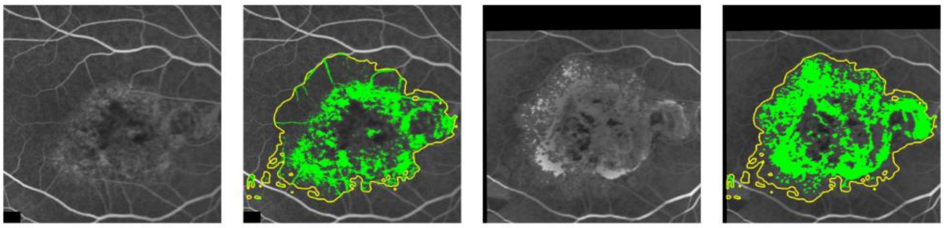

Pair ID : 9
Rank : 3
Date diff : 1834
Reference HA : 25913
Test HA : 39900
Mask : 91706
Related Fig : Fig. 5

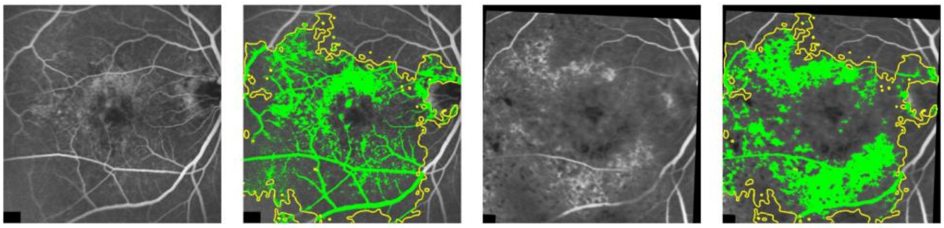

Pair ID : 11
Rank : 3
Date diff : 2055
Reference HA : 44610
Test HA : 49644
Mask : 153182
Related Fig : Fig. 5

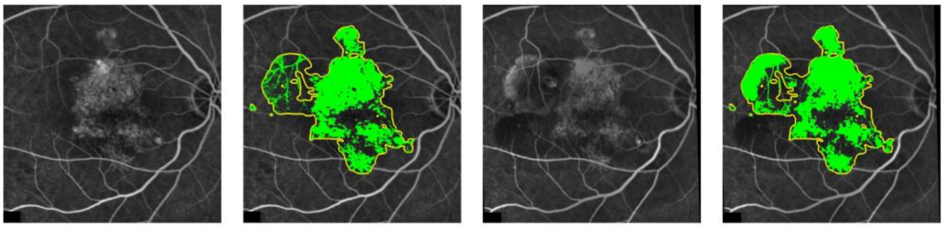

Pair ID : 15
Rank : 3
Date diff : 592
Reference HA : 27243
Test HA : 30241
Mask : 52032
Related Fig : Fig. 5

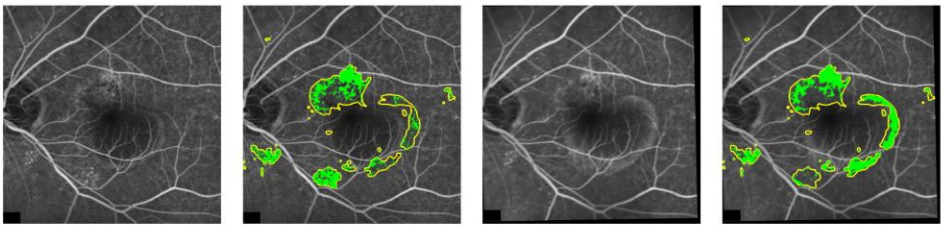

Pair ID : 16
Rank : 3
Date diff : 282
Reference HA : 7368
Test HA : 8701
Mask : 18355
Related Fig : Fig. 5

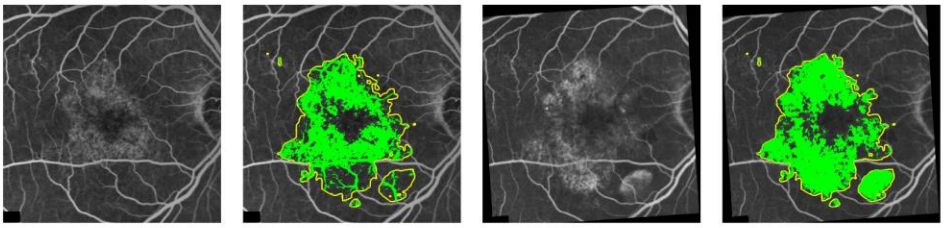

Pair ID : 19
Rank : 3
Date diff : 2215
Reference HA : 32390
Test HA : 40715
Mask : 61465
Related Fig : Fig. 5

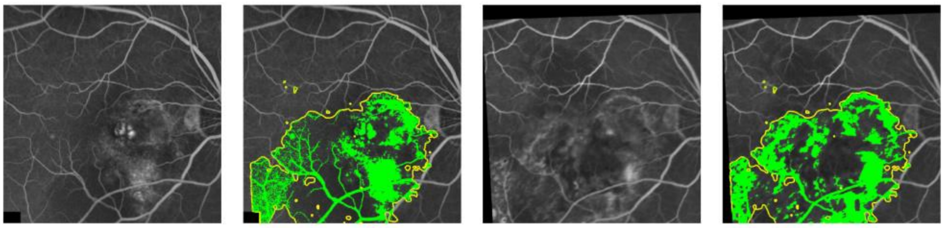

Pair ID : 20
Rank : 3
Date diff : 1682
Reference HA : 30153
Test HA : 35006
Mask : 86354
Related Fig : Fig. 5

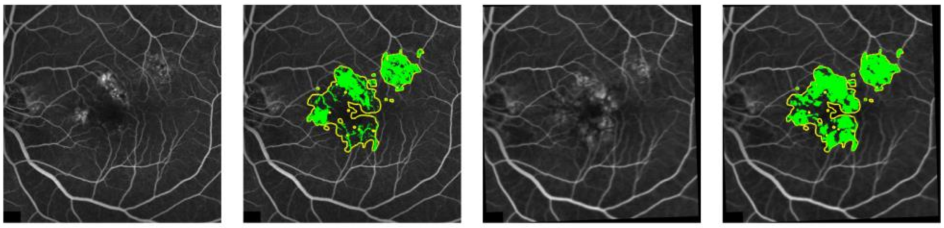

Pair ID : 55
Rank : 3
Date diff : 2572
Reference HA : 10110
Test HA : 15188
Mask : 23210
Related Fig : Fig. 5

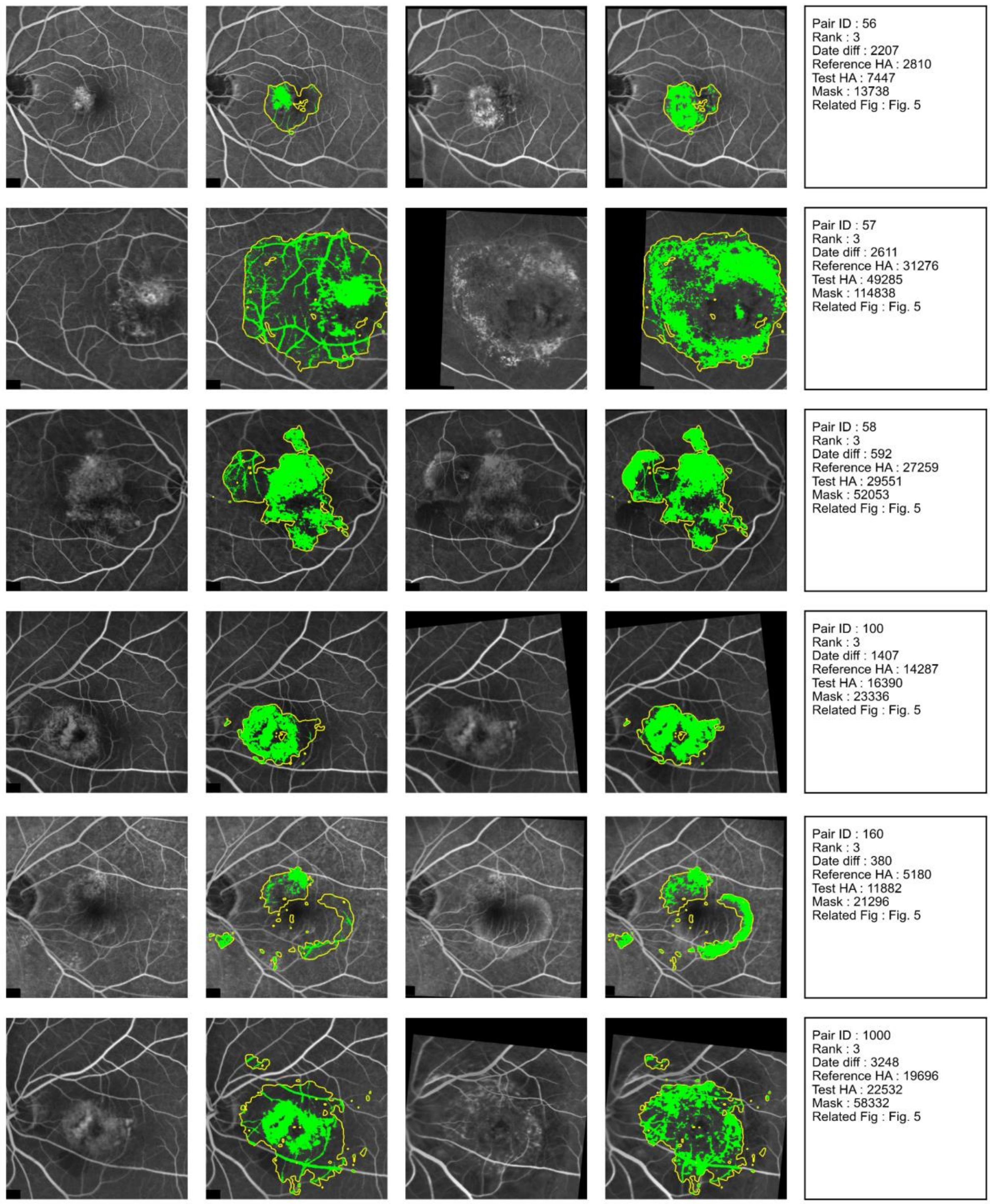

Figure S2. All pairs of images taken on different days

Two ophthalmologists unanimously judged 54 pairs (108 images) by 3 ranks.

(**a**), (**c**) A pair of aligned FA images, brightness-corrected by image processing.

(**b**), (**d**) A pair of fundus FA images with Predicted Abnormal regions (yellow) and Hyperfluorescent areas (green).

(**e**) "Pair ID" is a unique ID for each pair. "Rank 1~3" means that two ophthalmologists reviewed the images of the disease time course of each eye and unanimously judged the changes in the images at each time point as follows: "Rank 1," approximately the same, "Rank 2," slightly worse "Rank 3," much worse. "Date diff" is the difference between the acquisition dates of the two images. "Reference HA" is the Abnormal region output by (**a**) and (**b**), and "Test HA" is the Hyperfluorescent area output by (**c**) and (**d**). "Mask" is the area of the Abnormal region. "Related Fig." refers to the related one in the main Figure.
